# Cold-adapted carboxylesterases from Alcanivoracaceae active with a wide range of synthetic polyesters

**DOI:** 10.1007/s00253-026-13726-z

**Published:** 2026-02-07

**Authors:** Hairong Ma, Anna N. Khusnutdinova, Tatyana N. Chernikova, Manuel Ferrer, Alexander F. Yakunin, Olga V. Golyshina, Peter N. Golyshin

**Affiliations:** 1 Centre for Environmental Biotechnology, School of Environmental and Natural Sciences, Bangor University, Bangor, LL57 2UW UK; 2 Instituto de Catalisis y Petroleoquimica (ICP), CSIC, Madrid, 28049 Spain

**Keywords:** Carboxylestereases, Polyesters, Cold-adapted enzymes, Marine hydrocarbon-degrading bacteria, *Alcanivoracaceae*, *Alcanivorax*

## Abstract

**Abstract:**

Members of the family *Alcanivoracaceae* are widespread in marine environments, where they play central roles in hydrocarbon degradation and populate plastics-associated microbiomes, with notable enzymatic potential toward ester- and olefin-based polymers. To further investigate their enzymatic potential, we selected 21 candidate enzymes from the α/β-fold hydrolase superfamily, specifically carboxylesterase Family V from genome-sequenced representatives of the genera *Alcanivorax*, *Alloalcanivorax*, and *Isoalcanivorax*. Genes encoding enzymes were cloned and heterologously expressed in *E. coli*, of which eleven were purified and subjected to substrate specificity analyses including six previously reported and partially characterised carboxylesterases from *A.* *borkumensis* SK2, used as benchmarks. All enzymes showed activity against soluble model *p-*nitrophenyl ester substrates with acyl chain lengths ranging from C2 to C12 and against bis(benzoyloxyethyl) terephthalate (3PET) and polycaprolactone (PCL2). During 3PET hydrolysis, product accumulation followed the order: benzoic acid >  > MHET > terephthalic acid. Five enzymes hydrolysed polycaprolactone (PCL14), poly-DL-lactide (PDLLA), and polybutylene adipate (PBA). All five enzymes displayed temperature optima around or below 50 °C and retained high activity at low temperatures (5–20 °C), consistent with adaptation to marine environments. Enzymes also exhibited moderate solvent tolerance, neutral-to-alkaline pH optima, and low thermostability, with melting temperatures (*T*_m_) between 31 and 48 °C. Overall, enzymes from *Alcanivoracaceae* exhibited promising potential for synthetic polyesters’ biodegradation, especially under low-temperature conditions, suggesting potential application for degrading specific polyester-based plastics with lower molecular weight, and their utility in further enzyme engineering for plastic recycling and upcycling.

**Key points:**

• *Members of Alcanivoracaceae are a rich resource of polyester-degrading enzymes.*

• *All selected and analysed Family V esterases exhibited high activities and stabilities at low temperatures and solvent tolerance.*

• *Characterised enzymes were active with a broad range of polyesters.*

**Supplementary Information:**

The online version contains supplementary material available at 10.1007/s00253-026-13726-z.

## Introduction

Plastic pollution is an ever-escalating issue with detrimental effects on both the environment and human health. Over the years, plastics have become an indispensable part of daily life, serving critical roles in packaging, transportation, and healthcare. However, their widespread use has also led to a significant waste accumulation problem, making plastic pollution one of the greatest environmental challenges of the twenty-first century. Despite growing consumer awareness, the production of single-use plastics continues to escalate (Geyer et al. 2017; Stubbins et al. 2021). Among various strategies for addressing plastic waste, biological degradation, particularly enzymatic closed-loop recycling, has emerged as a promising solution (Abou-Zeid et al. 2004; Danso et al. 2019). For example, the first enzymatic polyethylene terephthalate (PET) degradation study was reported with a hydrolase (TfH) derived from *Thermobifida fusca* (Müller et al. 2005)*.* Over the past two decades, significant advancements in protein and process engineering have led to substantial improvements in the initially low PET degradation (PETase) activity of *T.* *fusca* (Barth et al. 2015; Müller et al. 2005; Roth et al. 2014). With the advancement of metagenomic approaches, more active enzymes, such as leaf-branch-compost cutinase LCC, have been identified (Sulaiman et al. 2012). Furthermore, protein engineering has enabled the development of mutant LCC variants (e.g. LCC_ICCG_, LCC_WCCG_) with enhanced and accelerated PET depolymerisation efficiency (Makryniotis et al. 2023; Tournier et al. 2020). Subsequently, a novel bacterium, *Ideonella sakaiensis* 201-F6, was isolated from plastic-contaminated sediment samples and found to exhibit both PETase and PET monoester-hydrolysing (MHETase) activities (Kan et al. 2021; Yoshida et al. 2016). IsPETase, initially identified and characterised in that study, has served as the foundation for numerous structural and protein engineering studies aimed at further enhancing its PET depolymerisation activity (Dai et al. 2021; Feng et al. 2021; Joo et al. 2018; Haugwitz et al. 2022; Wei et al. 2022).

Ongoing research efforts are focused on modifying the active sites of the wild-type enzymes to improve their catalytic efficiency, as well as engineering enzymes with enhanced thermostabilities through rational design and directed evolution (Bell et al. 2022; Shi et al. 2023; Schmidt et al. 2019; Williams et al. 2023). These advancements hold great potential for the development of efficient biocatalysts capable of supporting sustainable plastic waste management and recycling. Polyesterases, such as carboxylesterases, cutinases, lipases, and specific PETases, are particularly effective in degrading synthetic polyesters. Cutinases, found in both bacteria and fungi, hydrolyse the ester bonds in cutin and synthetic polyesters, leading to the formation of monomers such as terephthalic acid and ethylene glycol. PETases, which have gained considerable attention, are specialised enzymes capable of breaking down PET into its constituent monomers under mild conditions (Tournier et al. 2020). These enzymes have been found in various microorganisms, highlighting their potential for dual functionality in polyester degradation and upcycling (Yoshida et al. 2016).

Among bacteria, which hold significant potential for the degradation of polyesters, the genus *Alcanivorax*, a member of the family *Alcanivoracaceae* within the class *Gammaproteobacteria*, has attracted particular attention for over 25 years (Yakimov et al. 1998, 2022). This genus, along with the genera *Oleiphilus*, *Oleispira*, *Marinobacter*, and *Thalassolituus*, represents ubiquitous, truly marine bacteria primarily known to utilise hydrocarbons as their preferred source of carbon and energy (Staley 2010; Yakimov et al. 2004, 2022; Golyshin et al. 2002). Taxonomically, the genus *Alcanivorax* (family *Alcanivoracaceae*) was recently divided into three genera, including two new ones (Rai et al., 2023). Several species within this family exhibit a strong preference for aliphatic hydrocarbons, both linear and branched (Yakimov et al. 2022), with some *Alcanivoracaceae* species reported to degrade polycyclic aromatic hydrocarbons (PAHs) such as naphthalene and pyrene (e.g. *Alloalcanivorax xenomutans* SRM1 (previously known as *Alcanivorax xenomutans*)) (Dell’Anno et al. 2023) and aromatic hydrocarbons, e.g. xylene (*A.* *xenomutans* JC109) (Rahul et al. 2014). Many characterised strains do not assimilate sugars or amino acids but are capable of metabolising fatty acids, alcohols, and aliphatic hydrocarbons (Lai et al. 2011). Typically, *Alcanivoracaceae* species occur at low or undetectable levels in unpolluted environments; however, their growth is markedly stimulated by the presence of hydrocarbons (Yakimov et al. 2022). Given their ecological lifestyle, these organisms and their enzymes are expected to exhibit tolerance to organic solvents (Bollinger et al. 2020). Furthermore, *Alcanivoracaceae* species, along with other hydrocarbon-degrading taxa, were recently identified as frequent colonisers of surfaces of both polyesters- and olefin-based plastics in marine environments and were therefore considered potential degraders (Denaro et al. 2020; Delacuvellerie et al. 2019; Popovic et al. 2017; Tulloch et al. 2024; Zadjelovic et al. 2022).

In terms of its polyester-degrading capability, carboxylesterases from the type strain of the family, *Alcanivorax borkumensis* SK2^T^, have demonstrated broad substrate profiles. These enzymes are active not only against model substrates (i.e. *p*NP-esters of fatty acids with aliphatic chain lengths between C2 and C16), but also against synthetic (poly)esters such as PLLA, PDLLA, PCL, and 3PET (Hajighasemi et al. 2016, 2018; Tchigvintsev et al. 2015), indicating the broader biotechnological potential of this organism beyond its established role in the natural attenuation of oil spill pollution. Notably, such substrate promiscuity is common among carboxylesterases and has been extensively studied (Martínez-Martínez et al. 2018; Ma et al. 2025). Another species within the family, *Alloalcanivorax dieselolei* (previously known as *Alcanivorax dieselolei*), has been reported to encode a promiscuous, solvent-tolerant esterase (Zhang et al. 2014). Recent investigations of a strain isolated from plastic marine debris, *Alcanivora*x sp. 24 (recently renamed to *Alloalcanivorax* sp. 24), showed that its hydrolase ALC24_4107 exhibited a strong activity vs. aliphatic polyesters, such as PHB, PHBV, PES, PBS, and PCL (Zadjelovic et al. 2020). Of note, this very strain was also demonstrated to degrade the weathered low-density polyethylene (LDPE) by recruiting an array of redox enzymes (alkane monooxygenases, P450, laccases) and reactive oxygen species (Zadjelovic et al. 2022). As reported elsewhere, a promiscuous hydrolase from the metagenomic fragment of *Thalassolituus oleivorans* (another renowned marine oil-degrader) showed a hybrid ester hydrolase and haloacid-dehalogenase activity (Beloqui et al. 2010). As exemplified in this and other studies, mining metagenomes of petroleum-enriched microbial communities using activity-based screens is regarded as a highly productive approach to identify activities of yet uncharacterised enzymes and unknown proteins from hydrocarbon-based marine environments (Ferrer et al. 2005; Popovic et al. 2015, 2017).

This study aimed to identify novel polyesterases from *Alcanivoracaceae* species with the potential to degrade a wide range of synthetic esters, such as 3PET, PLLA, PDLA, PDLLA, PCL, PBA, PBS, and PC. While several esterases from *Alcanivoracaceae* species have been previously characterised, this research extends the investigation to other predicted hydrolases across a broader selection of strains from this family, including *Alloalcanivorax* sp. 24, *Alloalcanivorax gelatiniphagus*, and *Isoalcanivorax pacificus* (previously known as *Alcanivorax gelatiniphagus* and *Alcanivorax pacificus*, respectively). The genomes of these organisms encode a variety of putative α/β hydrolases and carboxylesterases, many of which remain uncharacterised and present exciting opportunities for further exploration. Notably, *Alcanivoracaceae* species are naturally adapted to marine environments, making them particularly well-suited for applications in low temperature conditions and energy-efficient industrial processes. Additionally, polyesterases have demonstrated considerable promise in various sectors, such as waste management, bioremediation, and the development of biodegradable plastics, further emphasising the relevance of this research for sustainable and environmentally friendly industrial practices.

## Materials and methods

### Chemicals

All chemicals and substrates used in this study were of analytical grade. Chromogenic *p*-nitrophenyl (*p*NP) esters (C_2_-C_16_) were purchased from Sigma-Aldrich/Merck (Gillingham, UK) and Tokyo Chemical Industry UK Ltd. (TCI, Oxford, UK). 6-hydroxycaproic acid (6-HHA), lactic acid (LA), succinic acid (SA), adipic acid (AA), terephthalic acid (TA, Cat no. 185361), bisphenol-A bis(2-hydroxyethyl) terephthalic acid (BHET, Cat no. 465151), Tween-20, dichloromethane (DCM), and 1,1,1,3,3,3-hexafluoro-2-propanol (HFP) were purchased from Sigma-Aldrich/Merck (Gillingham, UK).

Mono 2-hydroxyethyl terephthalic acid (MHET, Cat no. AMBH95E08A06) was sourced from Millipore Sigma (Ontario, Canada)/Ambeed Inc.). Polyester and oligomeric polyester model substrates, poly-D, L-lactide (PDLLA, molecular weight *M*_W_ 10,000–18,000, Cat no. 719978), polycaprolactone (PCL14, average *M*_W_ ~ 14,000, Cat no. 440752) (PCL2, *M*_n_ ~ 2000, Cat no. 189421), and amorphous polyethylene terephthalate (aPET, thickness 0.25 mm, coil width 600 mm, Cat no. ES30-FM-000145), polybutylene succinate (PBS, *M*_W_ 45,500, Cat no. 936340), poly-1,4-butylene adipate (PBA, *M*_W_ 12,000, Cat no. 181501), poly(1,4-butylene) terephthalate (PBT, *M*_W_ 38,000, Cat no. 190942), poly-L-lactic acid (PLLA, *M*_n_ 20,000, Cat no. 764698), poly-D-lactic acid (PDLA, *M*_n_ 20,000, Cat no. 767344), polycarbonate (PC, 3-mm granules, Cat no. GF65553598) were purchased from Sigma-Aldrich/Merck (Gillingham, UK). The PET model substrate, bis(benzoyloxyethyl) terephthalate (3PET, *M*_W_ 462.4), was synthesised by CanSyn (Toronto, Canada). Impranil® DLN was kindly donated by WhitChem Ltd, UK (Azelis, www.whitchem.co.uk). Isopropyl β-D-1-thiogalactopyranoside (IPTG), ampicillin, 2-(cyclohexylamino)ethanesulfonic acid (CHES), sodium chloride (NaCl), tris(hydroxymethyl)aminomethane hydrochloride (Tris-HCl), methanol, sulphuric acid, orthophosphoric acid, and Luria-Bertani (LB) broth (tryptone 10 g/L, yeast extract 5 g/L, sodium chloride 10 g/L) were also purchased from Sigma-Aldrich/Merck (Gillingham, UK).

### Gene cloning and protein purification

The coding sequences of selected hydrolase genes were commercially synthesised (without signal peptides, with the addition of N-terminal hexahistidine tags) and cloned into a modified p15TVL (TWIST BIOSCIENCE, South San Francisco, USA). All plasmids were transformed into the *E. coli* Lobstr BL21(DE3) cells (Kerafast, Boston, USA). *E. coli* cultures were grown aerobically in 2.5-L baffled Erlenmeyer flasks with 1 L Luria-Bertani medium supplemented with 4 g/L glycerol and 100 µg/ml ampicillin at 37 °C, in a shaking incubator at 200 rpm, to the optical density (OD_600_) 0.6–0.8, then spiked with 0.4 mM IPTG, transferred to 16 °C, and incubated at that temperature in a shaker for a further 16 h. The biomass was harvested by centrifugation at 4000 rpm for 10 min (Avanti J26 rotor JLA8.1, Beckman Coulter Life Sciences, Indianapolis, USA) and disrupted in an ice bath by sonication (Q-sonica, Newtown, USA) for 10 min at 70% intensity, in 4-s pulses with 5-s cooling time. Recombinant proteins were purified to near homogeneity (> 95%) using nickel-chelate affinity chromatography on Ni-NTA Superflow resin (QIAGEN, Hilden, Germany) as described previously (Distaso et al. 2023). Purity and protein size of purified enzymes were assessed using denaturing 10% polyacrylamide gel (BioRad Laboratories, Hercules, USA) electrophoresis, whereas protein concentration was measured by Bradford assay (Bio-Rad Laboratories, Hercules, USA).

### Carboxylesterase assays with soluble chromogenic substrates

The chromogenic *p*-nitrophenyl (*p*NP) esters of fatty acids with different acyl chain lengths: *p*NP-acetate (C2), *p*NP-butyrate (C4), *p*NP-hexanoate (C6), *p*NP-octanoate (C8), *p*NP-decanoate (C10), *p*NP-dodecanoate (C12), *p*NP-myristate (C14), and *p*NP-palmitate (C16) were used to test carboxylesterase activity of purified proteins. The activity was screened in 96-well plates spectrophotometrically using SpectraMax M3 (Molecular Devices, San Jose, USA). All assays were conducted in triplicates at indicated temperatures in 96-well plates with reaction mixtures (200 µl) containing 50 mM CHES (pH 9.0) buffer (or as indicated), 1 mM substrate, and 0.1–1 µg of enzyme. The reaction mixtures were incubated for 20 min at 30 °C, and the activity was calculated based on the absorbance of *p*-nitrophenol at 410 nm (*ε* = 17.8 mM^−1^ cm^−1^).

### Enzyme reaction conditions and stability determination

The effect of pH on carboxylesterase activity of purified proteins (pH profile) was determined using the universal Britton-Robinson buffer system (50 mM acetic acid, 50 mM phosphoric acid, 50 mM boric acid, pH range 4.0 to 10.5). Temperature optimum (temperature profile) for carboxylesterase activity of selected enzymes was measured at temperatures from 30 to 90 °C using 1 mM *p*NP-octanoate substrate and 0.1–1 µg of protein in 50 mM CHES buffer (pH 9.0). 200 µl assay reactions were incubated in a 96-well plate in the Thermomixer (Eppendorf, Hamburg, Germany) at 500 rpm. For the analysis of their thermal stabilities, selected proteins (1 mg/ml) were incubated for 2 h for thermostability and 5 h for cold stability (or as indicated) at different temperatures (from 5 to 90 °C) in the PCR thin wall tubes in T100 Thermal Cycler (Bio-Rad Laboratories, Hercules, USA), and the residual carboxylesterase activity was measured with 1 mM *p*NP-octanoate at 30 °C as described in the carboxylesterase assay methods section.

The effect of NaCl and Tween20 on carboxylesterase activity of selected proteins was analysed using 1.0 mM *p*NP-octanoate and 0.1–1 µg of enzyme. In the reaction mixture of 200 µl containing NaCl at concentrations between 0.1 and 2.0 M and Tween-20 in the range of 0.1–3.0% in 50 mM CHES, pH 9.0, reaction mixtures were incubated for 20 min at 30 °C, and the activity was measured spectrophotometrically at 410 nm.

The effect of organic solvents on carboxylesterase activity was analysed in the conditions described in the carboxylesterase assays with soluble chromogenic substrates section, but supplied with various concentrations of dichloromethane, dimethylsulfoxide, ethanol, and 1,1,1,3,3,3-hexafluoro-2-propanol in the range between 0 and 50%, in 50 mM CHES, pH 9.0.

All assays were carried out in triplicates.

### Analysis of temperature-dependent protein denaturation

The melting temperature of selected enzymes was measured by differential scanning fluorimetry (DSF) on a QuantStudio 6 Flex system (Applied Biosystems, Thermo Fisher Scientific, Waltham, USA) using SYPRO Orange (Invitrogen, Carlsbad, USA) as a binding dye. Experiments were conducted in triplicate in 30 µl reaction volumes with 10 µg of protein in 50 mM CHES buffer (pH 9.0) and 25× SYPRO Orange in sealed optically clear 96-well plates (Applied Biosystems, Thermo Fisher Scientific, Waltham, USA) with the system set on the ROX as the reference wavelength using the 450/490 nm excitation and 560/580 nm emission filters. The temperature was increased from 25 to 95 °C with an increment of 1 °C s^−1^. *T*_m_ values for selected proteins were determined by non-linear fitting of the Sigmoidal-Boltzmann equation using the GraphPad Prism software (version 5.0).

### Preparation of polyester substrates and polyesterase assays

Polyesters and oligomeric polyester model substrates used in this study (PDLLA, PLLA, PDLA, PCL14, PCL2, 3PET, PBS, PBA, PBT, aPET) were prepared in 50 mM Tris-HCl (pH 8.0), as described previously (Distaso et al. 2023). For agarose-based screens, 0.5% polyester emulsions were diluted with three volumes of CHES buffer (pH 9.0) in 2% (w/vol) molten agarose, poured, and solidified in the round 90 mm Petri dishes to make opaque gel with the final concentration of polyesters 0.125% (w/vol). Impranil® DLN was used only for agarose gel plate preparation. For this, 1 ml Impranil DLN was mixed with 100 ml 50 mM CHES (pH 9.0) and molten agarose, with the final concentration of Impranil 1% in 2% (w/vol) agarose. Fifty micrograms of enzyme aliquots was loaded into cylindrical wells cored in the agarose using cut 1-ml micropipette tips. Plates were placed into plastic bags to prevent evaporation, incubated at 30 °C, and monitored for 2–3 days. The formation of a clear zone around the wells was considered an indication of the presence of polyester-degrading activity. Each enzyme was analysed in a single experiment under identical conditions.

For polyesterase assays in solution, the 200-µl reaction mixtures with 50 µg of purified protein and 0.125% of emulsified polyester in 50 mM CHES buffer (pH 9.0) were incubated for 12 h at 30 °C in a shaker at 500 rpm. The reactions were spun down at 14,000 g for 10 min in a minicentrifuge, and supernatant was filtered through 10-kDa spin filters (14,000 g, 15 min, Eppendorf 5424 centrifuge, Eppendorf AG, Hamburg, Germany). The presence of polyester degradation products in filtrates was analysed using HPLC Prominence-I LC-2030C 3D Plus equipped with a UV-VIS detector (Shimadzu, Kyoto, Japan). For aPET, 3PET, and PBT, the formation of terephthalic acid (TA), mono-2-hydroxyethyl terephthalic acid (MHET), and bis-2-hydroxyethyl terephthalic acid (BHET) was analysed. For PC depolymerisation, the bisphenol-A (BPA) was analysed. For product analysis, reverse-phase hydrophobic chromatography on a Shimadzu C_18_ Shim-pack column (150 × 4.6 mm, 5 µm particle size) (Shimadzu Corporation, Kyoto, Japan), 40 °C (injection volume 10 µl, detection at 240 nm) was used. For product separation, we used the mobile phase of Solvent A (0.1% (vol/vol) of orthophosphoric acid (H_3_PO_4_) in HPLC grade water) and Solvent B (100% methanol). At a flow rate of 0.7 ml/min, 40 °C, the gradient was as follows: 0–2 min, 25% Solvent B; 2–18 min, linear gradient to 55% of Solvent B; 18–22 min, linear gradient to 25% of Solvent B. For PDLA, PLLA, and PDLLA depolymerisation efficiency, lactic acid was quantified; for PCL14 and PCL2, 6-hydroxyhexanoic acid was quantified; for PBS depolymerisation, succinic acid was quantified; for PBAT, adipic acid was quantified using ion-moderated partition chromatography on a Prominence-I LC-2030C 3D Plus HPLC system equipped with an Aminex HPX-87-H column (300 × 7.8 mm, 9 µm particle size, conditioned at 50 °C) (Shimadzu Corporation, Kyoto, Japan) and a UV detector (LC-2030C_3Dplus, Shimadzu Corporation, Kyoto, Japan). For analysis, 0.05 N sulphuric acid (H_2_SO_4_) in HPLC grade water was used as solvent at a flow rate of 0.6 ml/min, detection at 210 nm. (Poly)ester degradation products were quantified based on their calibration curves generated using commercially available standards (TA, MHET, BHET, LA, 6-HHA, AA, SA, BPA). All assays were carried out in triplicate.

### Bioinformatic and structural analyses

Multiple sequence alignments of selected carboxylesterases were performed using the MAFFT online service, automated regime (Katoh et al. 2019). Alignment was visualised and modified using the online software STRAP (Gille et al. 2014). For phylogenetic analysis, five genomes of *Alcanivoracaceae* strains were screened for IPR029058 (AB hydrolase) signature containing sequences, and the 297 sequences were retrieved and aligned in Geneious Prime using global alignment with free end gaps, Cost matrix Blosum 62, and FastTree was used for phylogeny analysis. Structural analysis, surface charge, and hydrophobicity analysis were performed in UCSF ChimeraX 1.14 software (Meng et al. 2023). Protein structures were predicted using AlphaFold2 via the ColabFold Google Colaboratory implementation with default parameters, using MMseqs2-based multiple sequence alignment and model selection based on pLDDT scores.

## Results

### Analysis of Alcanivoracaceae genomes for potential carboxylesterases and novel polyesterases

**Fig. 1:**
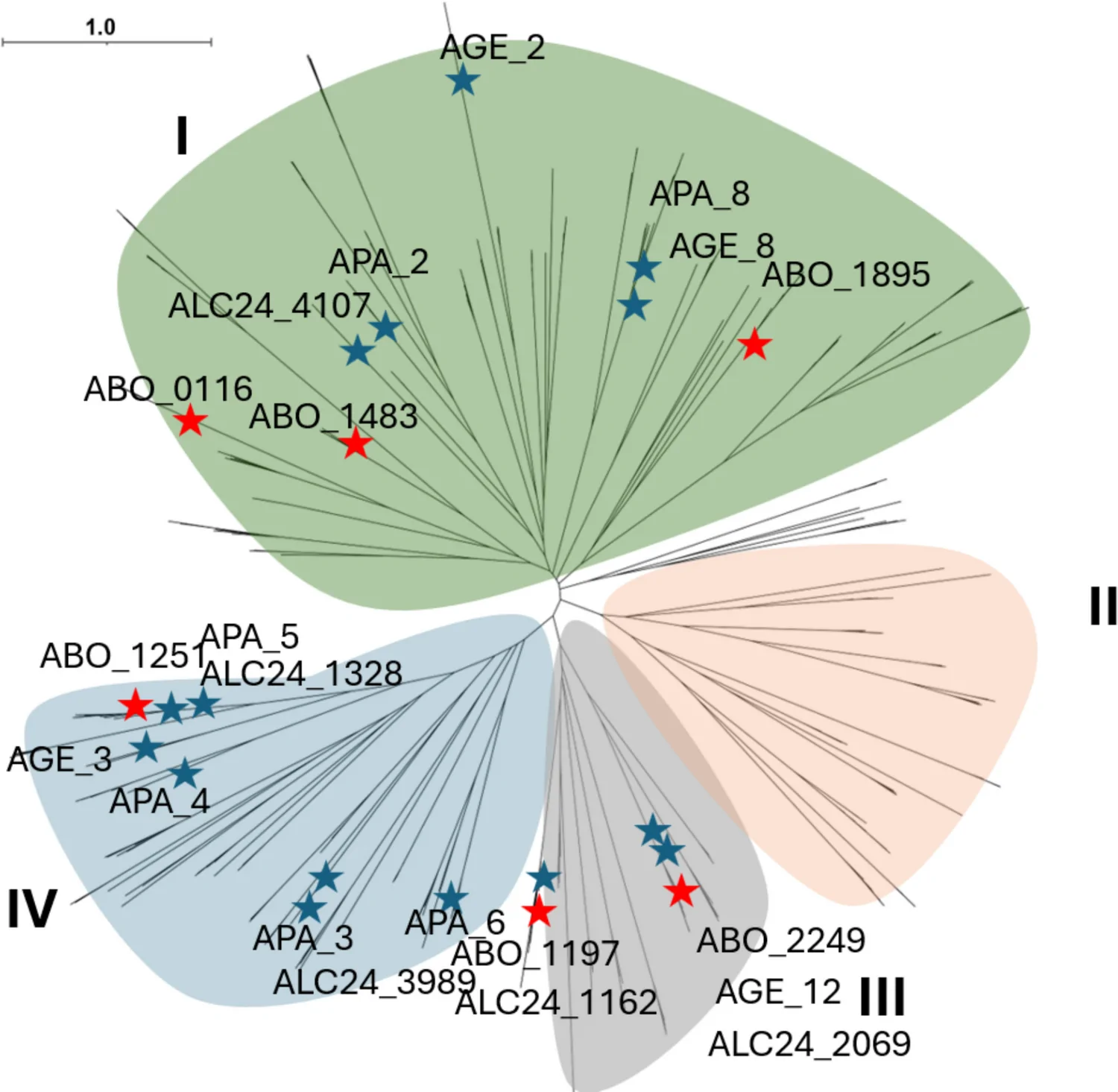
Maximum likelihood phylogenetic tree of 297 α/β hydrolase sequences from the genomes of *Alcanivoracaceae* type species, including Alcanivorax borkumensis SK2^T^, Alcanivorax hongdengensis A-11-3^T^, Alcanivorax sediminis PA15-N-34^T^, Isoalcanivorax pacificus W11-5^T^, Alcanivorax profundi MTEO17^T^, Alcanivorax nanhaiticus 19-m-6^T^, Alcanivorax gelatiniphagus MEBiC08158^T^ and Alloalcanivorax sp. 24, all containing the IPR029058 (AB hydrolase) signatures. Red stars indicate previously published proteins (ABO_0116, ABO_1483, ABO_1895, ABO_1251, ABO_1197, and ABO_2249), while blue stars denote proteins identified in this study (AGE_2, AGE_3, AGE_8, AGE_12, APA_2, APA_3, APA_4, APA_5, APA_6, APA_8, ALC24_1162, ALC24_3989, ALC24_1328, ALC24_2069, and ALC24_4107). The scale bar represents one substitution per position

In this study, we used five reference *Alcanivoracaceae* genomes (*Alcanivorax borkumensis* SK2^T^, *A.* *hongdengensis* A-11-3^T^, *A.* *sediminis* PA15-N-34^T^, *A. profundi* MTE017^T^, and *A.* *nanhaiticus* 19-m-6^T^), for screening of IPR029058 (ɑ/β hydrolase) signature containing sequences. A total of 297 sequences were retrieved from GenBank, 5 sequences manually selected from *Isoalcanivorax pacificus* previously known as *Alcanivorax pacificus* (APA_2, APA_3, APA_4, APA_5, APA_6), 4 sequences (AGE_2, AGE_3, AGE_8, AGE_12) selected from *Alloalcanivorax gelatiniphagus* originally named *Alcanivorax gelatiniphagus*, 6 sequences (ABO_0116, ABO_1197, ABO_1251, ABO_1483, ABO_1895, ABO_2249) from *A.* *borkumensis*, 5 sequences (ALC24_3989, ALC24_1328, ALC24_1162, ALC24_2069, ALC24_4107) from *Alloalcanivorax* sp. 24 (SI Table 1) and added for multiple sequence analysis (SI Fig. 1). Selected esterases from *Alcanivorax borkumensis* (ABO_0116, ABO_1197, ABO_1251, ABO_1483, ABO_1895, and ABO_2249) were partially characterised in previous studies (Hajighasemi et al. 2016, 2018; Tchigvintsev et al. 2015), which are highlighted in the phylogenetic tree (Fig. 1).

Analysis of enzymes using SignalP 5.0 (Almagro Armenteros et al. 2019) revealed that ABO_1197, ABO_1895, APA_6, and APA_5 have signal peptides (S1 Table 1), suggesting that the majority of *Alcanivoracaceae* esterases are involved in the intracellular metabolism. Selected sequences shared identity from 4.6 to 66.5% with characterised enzymes from *Alcanivorax borkumensis* SK2 (ABO_0116, ABO_1197, ABO_1251, ABO_1483, ABO_1895, and ABO_2249); their phylogenetic relatedness is presented in the SI Fig. 2, and according to the Arpigny and Jaeger (1999) classification of lipolytic enzymes, belonged to the carboxylesterase family V with the characteristic catalytic Ser motif GX**S**XGG (SI Fig. 1). Based on the sequence analysis, eight proteins (APA_2, ALC24_4107, AGE_2, APA_8, AGE_8, ABO_1898, ABO_0116, ABO_1483) belong to family V, subfamily I, sharing serine motif with GX**S**XG sequences. There were 5 sequences referred to family V subfamily III (AGE_12, ALC24_2069, ALC24_1162, ABO1197, ABO2449), sharing serine motif with GX**S**GG, and the rest 8 sequences belonging to family V subfamily IV (APA_5, ALC24_1328, AGE_3, APA_4, ALC24_3989, APA_3, APA_6, ABO1251), with the serine motif GX**S**XGX (Fig. 1).

**Fig. 2:**
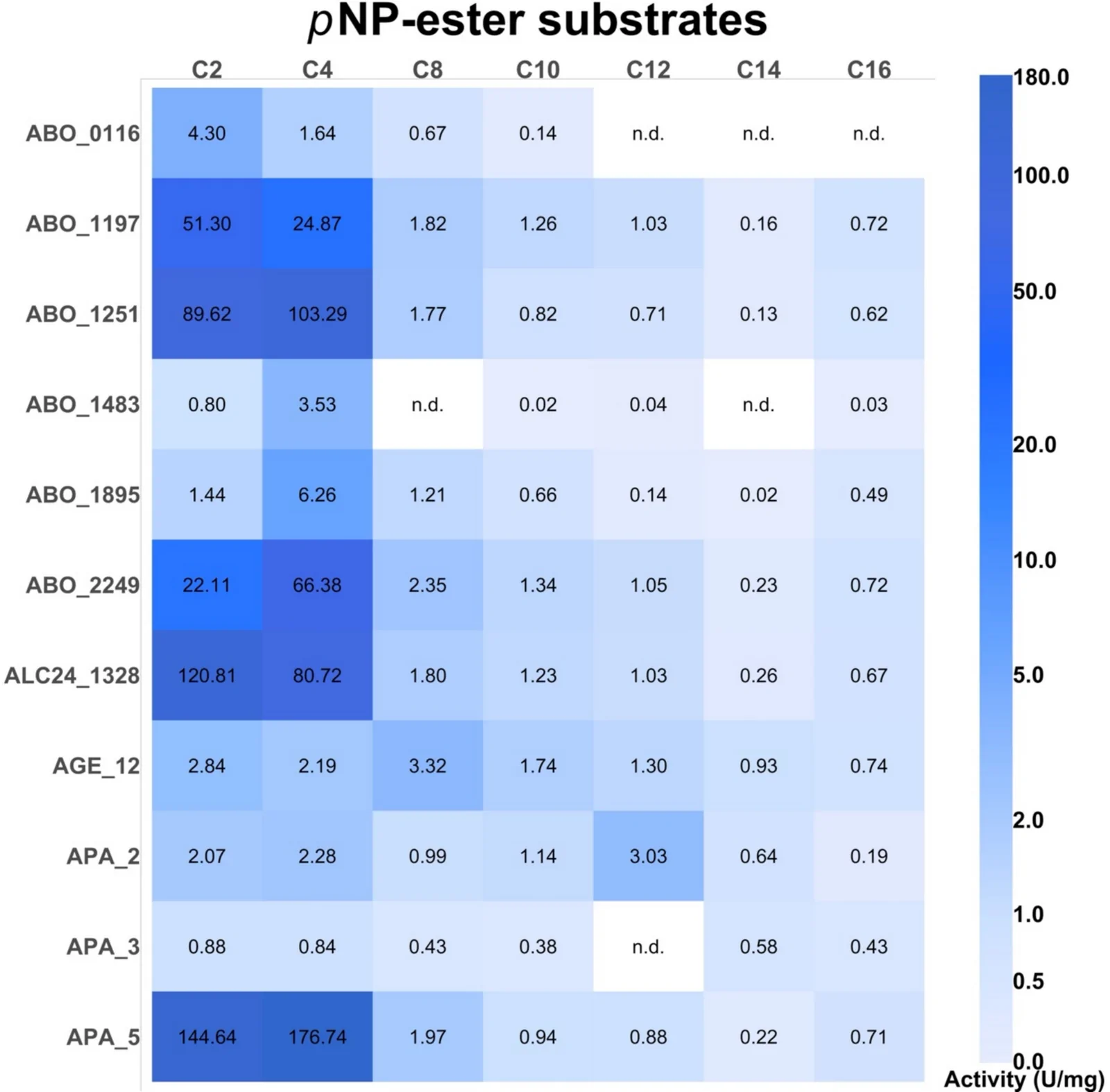
Assays of α/β-hydrolases for carboxylesterase activity using chromogenic *p*NP-esters with varying acyl chain lengths (C2–C16). Activities were measured in a reaction mixture with 1 µg of enzyme in 50 mM CHES (pH 9.0) and the specified substrates (1 mM) at 30 °C for 20 min. All assays were conducted in triplicates. Mean and standard deviation (SD) values are provided in the SI Table 4

### Carboxylesterase activity against chromogenic monoester substrates

The selected 21 genes encoding ${\alpha }/\upbeta$-hydrolases and predicted carboxylesterases (S1 Table 1) were recombinantly expressed in *E. coli* with an N-terminal 6His-tag and affinity purified (SI Fig. 3). Eleven proteins were found to be expressed soluble and subjected to further detailed analysis (AGE_12, APA_2, APA_3, APA_5, ALC24_1328, ABO_0116, ABO_1197, ABO_1251, ABO_1483, ABO_1895) (SI Fig. 3A). These 11 purified esterases were initially screened at 30 °C using a range of chromogenic *p*NP-esters with varying acyl chain lengths from C2 to C16 (Fig. 2, SI Table 4). These screens revealed the presence of carboxylesterase activity in all purified enzymes, with most enzymes showing the highest activity with short-chain esters (*p*NP-acetate and -butyrate) and low activity against longer acyl chain esters. In agreement with previous studies (Hajighasemi et al. 2016, 2018; Tchigvintsev et al. 2015; Urbanek et al. 2020), ABO_0116, ABO_1197, ABO_1251, ABO_1895, and ABO_2249 showed the preference to the short chain length substrates. From five novel *Alcanivoracaceae* proteins (AGE_12, APA_2, APA_3, APA_5, and ALC24_1328), APA_5 and ALC24_1328 exhibited the highest carboxylesterase activity with the C2 and C4 substrates, whereas the remaining three (AGE_12, APA_2, and APA_3) showed low activity (Fig. 2, SI Table 4). ALC24_1163 did not reveal any significant activity with chromogenic substrates.

**Fig. 3:**
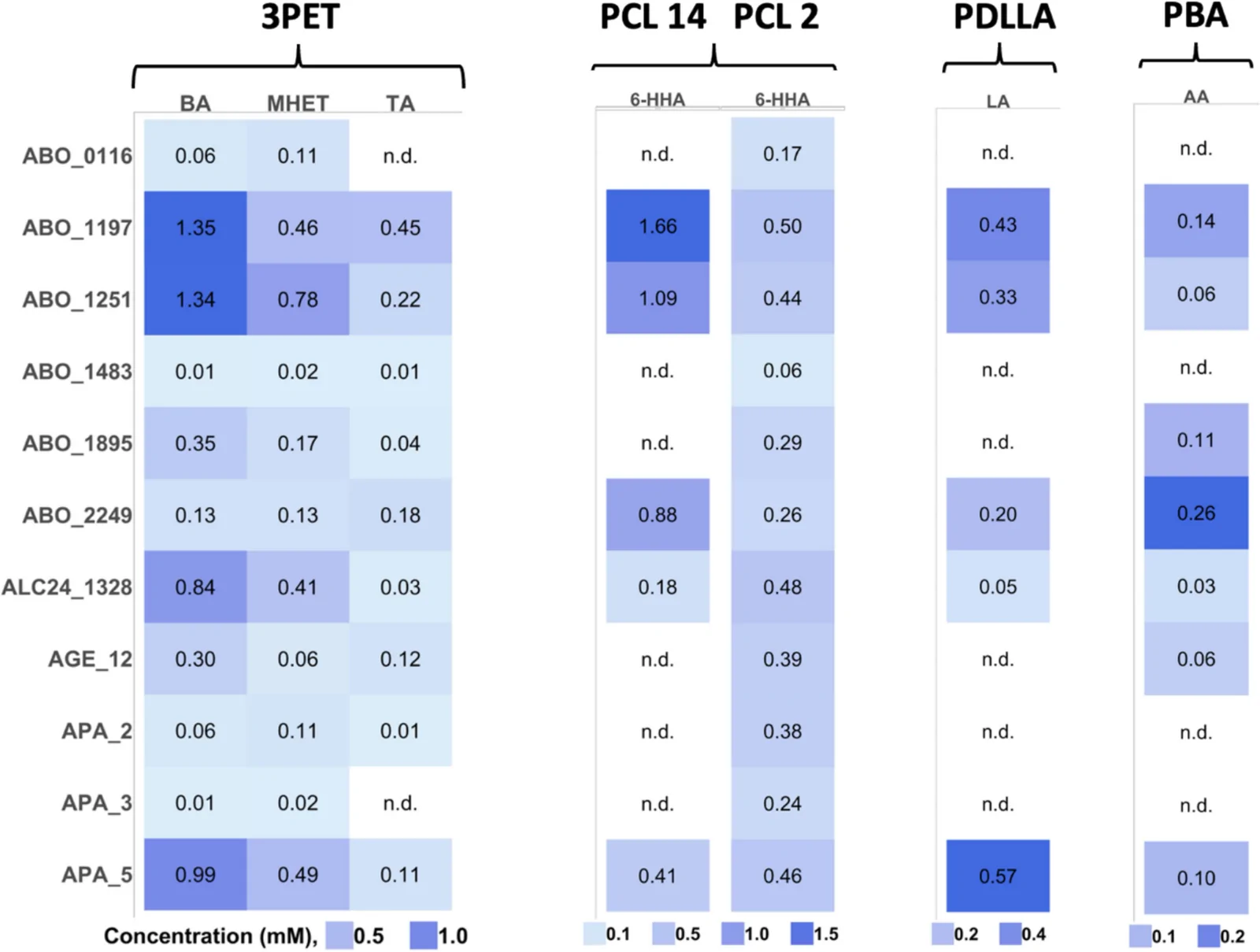
Polyesterase activities of selected carboxylesterases as revealed by reactions products analyses using HPLC. Fifty micrograms of enzymes was incubated with emulsified polyesters at 30 °C for 16 h, and the indicated reaction products were analysed by HPLC, as described in the ‘Materials and methods’ section. All assays were carried out in triplicate. ‘n.d.’, not detectable (below the detection limit of 0.01 mM)

### Activity of carboxylesterases against different polyesters

Eleven purified carboxylesterases from *Alcanivoracaceae* were then screened for depolymerisation activity against various polyesters, including 3PET, aPET, PCL14, PCL2, PDLA, PLLA, PDLLA, PBA, PBS, PBT, PC, and Impranil DLN. Assays were conducted using both the agarose plate-based screens and HPLC analyses of soluble reaction products after enzyme incubation with plastic emulsions. Visual inspection of agar plates revealed halos only around ABO_2249 and ABO_1197 for 3PET or PCL2. ABO_2249 and ABO_1197 were identified as active on the PBA emulsion agar plate (SI Fig. 4). However, no polyesterase activity against aPET, PLLA/PDLA, PDLLA, PBT, or PBS was detected in agarose plate screens. HPLC assays (Fig. 3 and SI Table 2) appeared more sensitive and revealed even slight depolymerisation not detected by agar plate visual inspection. Thus, hydrolytic activity with short-chain polyester oligomers like 3PET or PCL2 was observed for all esterases (Fig. 3), indicating enzymes’ broad esterase substrate spectra and catalytic promiscuity and their potential for plastic-degrading activity. Furthermore, the HPLC analysis was performed to detect soluble reaction products following incubation of purified enzymes with emulsified polyester substrates aPET, PCL14, PDLA, PLLA, PDLLA, PBA, PBT, and PC. These assays followed the formation of various polyester degradation products, such as TA, MHET, and BA from 3PET, 6-HHA from PCL14 and PCL2, LA from PDLA, PLLA, and PDLLA; AA from BPA, SA from PBS, TA from PBT, and BPA from PC. In these experiments, positive results were observed for PCL14, PCL2, PDLLA, PBA, and 3PET as substrates, but no polyesterase activity was detected against aPET and PBS, PDLA, PLLA, or PC (SI Table 2).

**Fig. 4:**
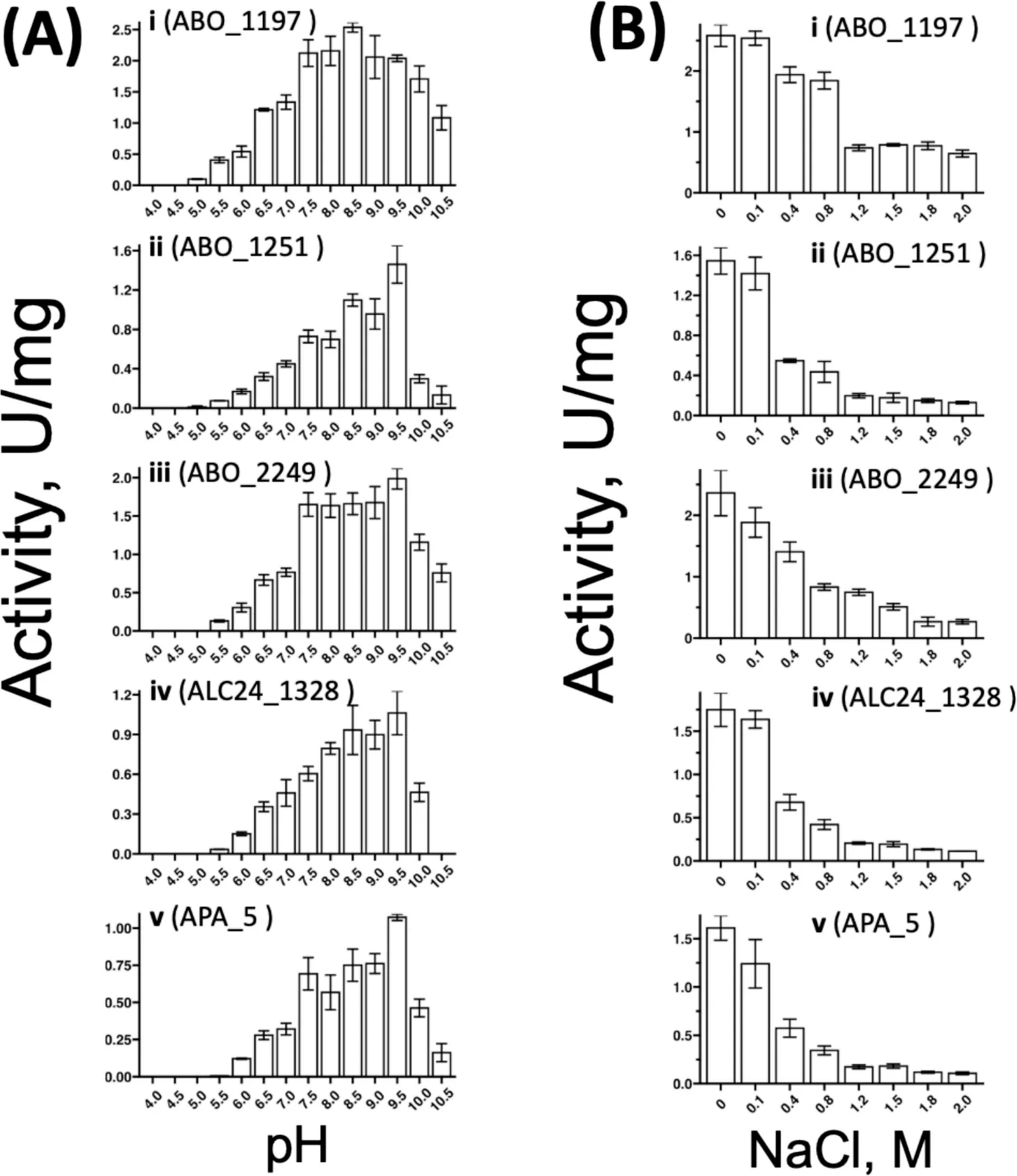
Effect of **A** pH and **B** NaCl concentrations on carboxylesterase activity of selected esterases with soluble chromogenic substrates. **A** Reactions were carried out in Britton–Robinson buffer over a pH range of 4.0–10.5 using 1 mM *p*NP-octanoate as substrate and 1 µg of enzyme per reaction, incubated for 20 min at 30 °C. **B** The effect of NaCl was tested in the concentration range of 0–2 M in 50 mM CHES buffer (pH 9.0) using 1 mM *p*NP-octanoate and 1 µg of enzyme per reaction (incubation for 20 min at 30 °C). All assays were carried out in triplicate

Interestingly, the major product during 3PET depolymerisation was MHET, with minor amounts of TA, and none of them producing BHET, indicating that the selected enzymes possess high BHETase activity and low MHETase activity (Fig. 3). ABO_1197, ABO_1251, ALC24_1328, and APA_5 exhibited high activity against 3PET, generating significant amounts of BA, MHET, and TA (from 0.2 to 1.35 mM). In contrast, ABO_0116, ABO_1483, ABO_1895, ALC24_1328, and APA_3 showed low activity, producing little to no BA, MHET, or TA as products (< 0.1 mM). Surprisingly, the highest accumulation of MHET (at 0.78 mM) was observed for ABO_1251. Since this enzyme produced no visible halos on the 3PET-agarose plates, this discrepancy highlights the higher precision of HPLC analysis for quantifying the depolymerisation activity.

With shorter-chain substrate PCL2, all tested *Alcanivoracaceae* enzymes were active, generating 6-hydroxyhexanoic acid (6-HHA) at concentrations ranging from 0.1 to 0.5 mM. However, only five enzymes (ABO_1197, ABO_1251, ABO_2249, ALC24_1328, and APA_5) exhibited activity against the longer-chain PCL14, producing 6-HHA in the range 0.2–1.7 mM (SI Table 2). A similar trend was observed with PDLLA (poly-D,L-lactide, MW range 10–18 kDa), where ABO_1197, ABO_1251, ABO_2249, APA_5, and ALC24_1328 produced significant levels of lactic acid (from 0.1 to 0.6 mM) (SI Table 2). The PDLLA polymer consists of a racemic mix of L- and D-lactic acid monomers, making it less crystalline than PLLA (L-lactic acid polymer) or PDLA (D-lactic acid polymer) (Lim et al. 2008), which may facilitate its enzymatic degradation. For PBA degradation, six enzymes (ABO_1197, ABO_1251, ABO_1895, ABO_2249, AGE_12, APA_5) demonstrated hydrolytic activity, producing adipic acid at concentrations ranging from 0.1 to 0.3 mM (SI Table 2).

In conclusion, based on their promising activities, five esterases, ABO_1197, ABO_1251, ABO_2249, APA_5, and ALC24_1328 were selected for further characterisation.

### Analysis of optimal reaction conditions for polyester depolymerisation

To identify the potential industrial applications of enzymes, they need to be screened for several key parameters, including substrate specificity, thermal stability to withstand industrial processing temperatures, pH stability across various industrial conditions, catalytic efficiency, and resistance to potential inhibitors present in industrial environments.

The selected enzymes ABO_1197, ABO_1251, ABO_2249, APA_5, and ALC24_1328 were screened for their optimal conditions regarding pH, NaCl detergent (Tween20) concentrations, and temperature. All selected enzymes preferred alkaline conditions (pH 8.0–9.0) (Fig. 4A). Salinity tolerance demonstrated that ABO_1197, ABO_1251, and ALC24_1328 maintained over 90% of activity at concentrations up to 0.1 M NaCl, while ABO_2249 and APA_5 generally exhibited lower salt tolerance. Notably, ABO_1197 showed the highest residual activity at 2 M NaCl, retaining 23% of its initial activity (Fig. 4B).

**Fig. 5:**
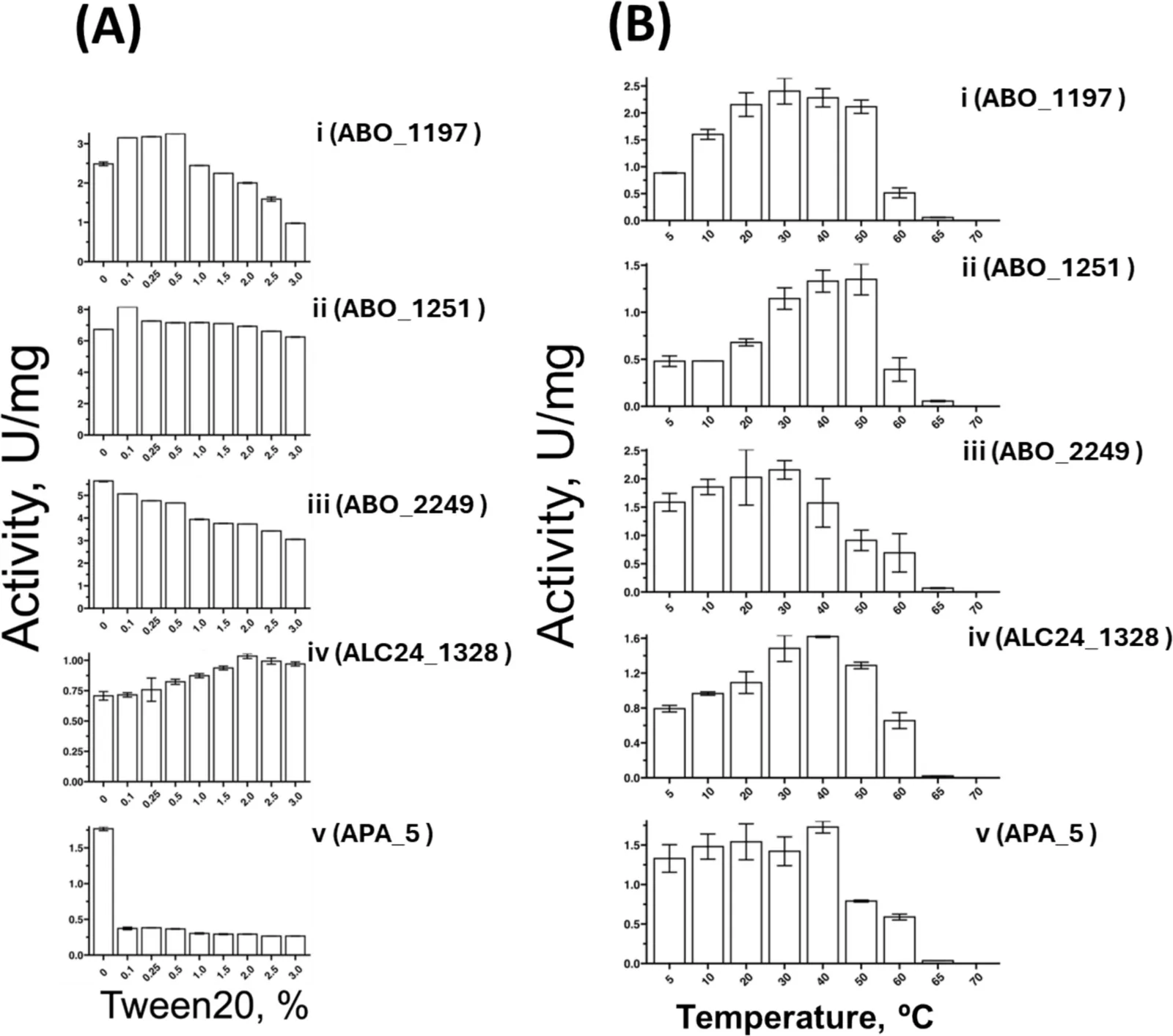
Effect of **A** Tween 20 and **B** reaction temperatures on carboxylesterase activity of selected enzymes with soluble chromogenic substrates. **A** The effect of Tween 20 was tested in the concentration range of 0–3% (v/v) in 50 mM CHES buffer (pH 9.0) using 1 mM pNP-octanoate as substrate and 1 µg of enzyme per reaction, incubated for 20 min at 30 °C. **B** The effect of reaction temperature was tested in the range of 5–70 °C in 50 mM CHES buffer (pH 9.0) using 1 mM pNP-octanoate and 1 µg of enzyme per reaction, incubated for 20 min. All assays were carried out in triplicate

For detergent tolerance, the non-ionic surfactant Tween20 was used, ranging at concentrations from 0.1 to 2.0%. ABO_1197 and ABO_1251 showed increased activity with Tween20, which is often attributed to enhanced substrate accessibility. However, ABO_2249 and APA_5 experienced a decrease in activity even with small traces of detergent, while ALC24_1328 displayed remarkable detergent resilience, increasing its activity by up to 25% at 2.0% Tween20 (Fig. 5A).

Temperature profiles were assessed from 5 to 70 °C, with all selected enzymes showing mesophilic traits, favouring temperatures between 10 and 50 °C. Most enzymes lost two-thirds of their activity at 60 °C. ABO_2249 and APA_5 demonstrated notable cold tolerance, with activities at 5 °C being just 24% less than at their respective optima (Fig. 5B).

### Analysis of thermostability

In the literature, protein stability is commonly assessed by measuring the melting temperature (*T*_m_), which is defined as the temperature at which equal amounts of the protein are folded and unfolded under specific conditions (Huynh and Partch 2015; Sorgenfrei et al. 2024). All applicable methods share the same principle: a sample is gradually heated, protein stability is monitored by melting temperature, which is either measured by the fluorescence signal of internal tryptophan residues or by using a fluorescence dye that changes its signal upon interacting with the unfolding protein. In this study, we investigated the thermostability of purified polyesterases by analysing the remaining enzyme activity after preincubation at different temperatures, as well as by identifying protein melting temperatures (*T*_m_) using DSF.

**Fig. 6:**
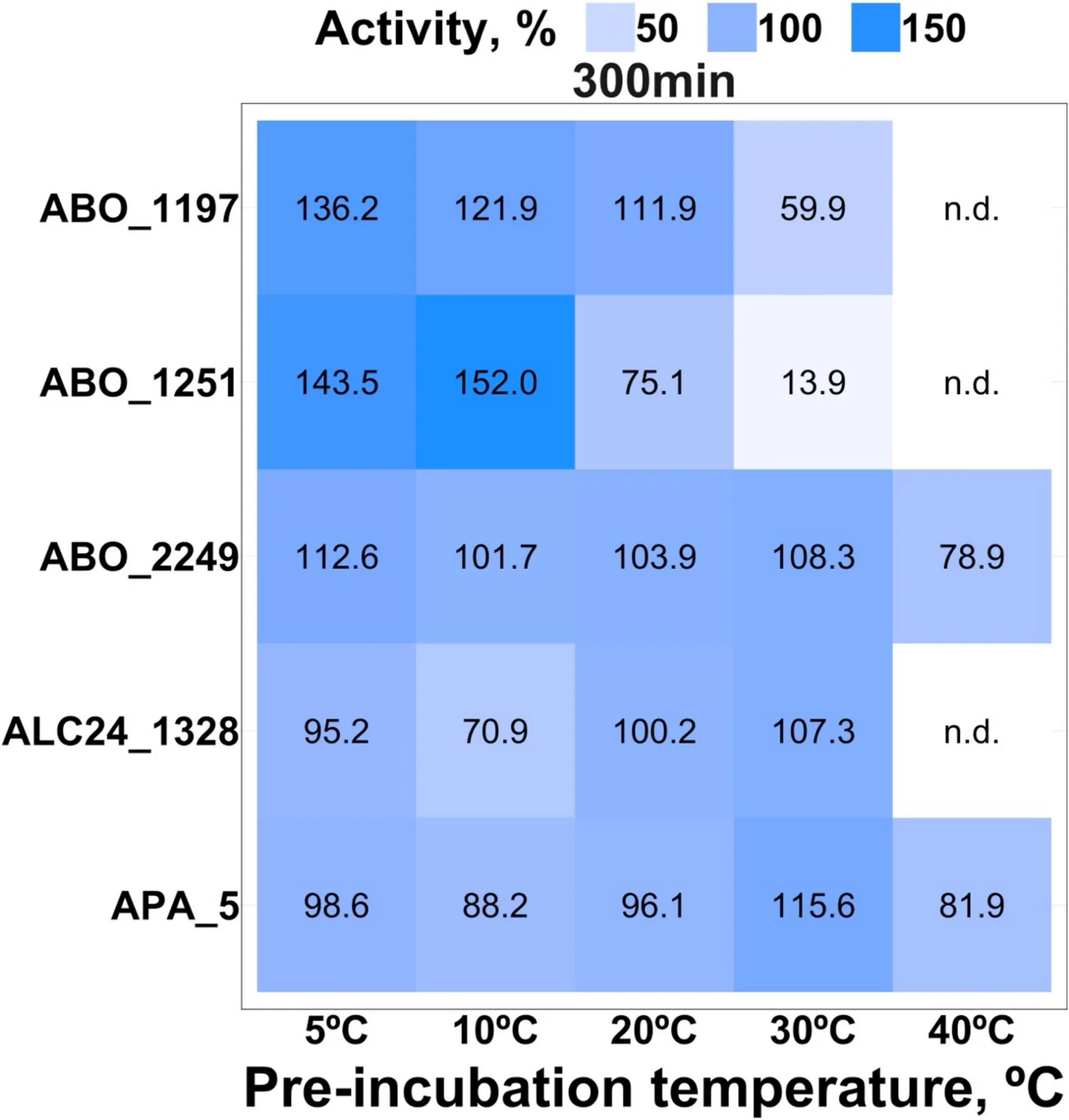
Cold tolerance of purified polyesterases: activity-based analysis. Following 5 h pre-incubation at different temperatures from 5 to 40 °C, residual carboxylesterase activities were measured at 20 °C with 1 mM *p*NP-octanoate as substrate, 1 µg of enzyme per reaction in 50 mM CHES buffer at pH 9.0. ‘n.d.’, not detectable (below the detection limit of 0.01 mM). All assays were carried out in triplicate

As shown in Fig. 6, after 5 h of preincubation at temperatures ranging from 5 to 40 °C, the polyesterases retained almost 100% of their initial activity at 5 °C. ABO_1197, ABO_1251, and ABO_2249 exhibited increased activity at 5 °C and 10 °C; however, ABO_1197 and ABO_1251 lost 40% and 80% of their activity at 30 °C and completely lost it at 40 °C. APA_5 and ALC24_1328 showed only a slight (2–5%) decrease in activity after incubation at 5 °C. Overall, the selected enzymes maintained good activity within the low-temperature range (5–30 °C) over incubation, with a maximum tolerance temperature of 30 °C.

Protein melting temperatures (*T*_m_) were determined based on DSF and are shown in SI Fig. 5. The highest melting temperature was observed for APA_5 (*T*_m_ 48.1 °C), correlating with its high residual activity at 30 °C after 5 h of incubation (Fig. 6).

### Analysis of solvent resistance

Organic solvent tolerance of enzymes was determined by measuring activity in the presence of solvents concentrations varying from 0 to 50%. Four different water-miscible organic solvents were chosen: ethanol, DMSO, dichloromethane (DCM), and 1,1,1,3,3,3-hexafluoro-2-propanol (HFP) based on their relevance for synthetic organic chemistry and for synthetic polymers. DCM and HFP are commonly used to dissolve the polyesters and were used for emulsions preparation in this work.

**Fig. 7:**
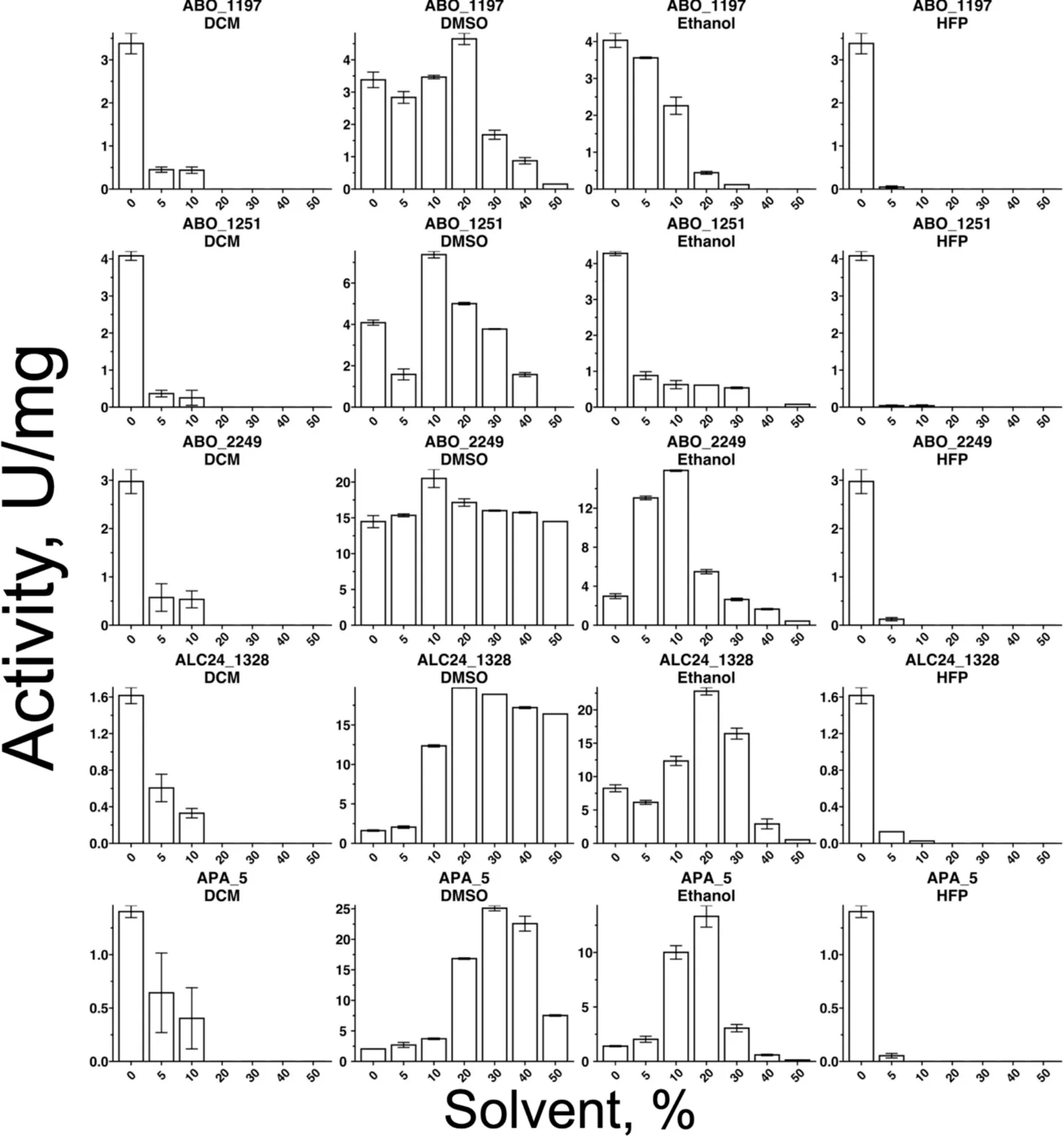
Effects of various organic solvents at concentrations from 0 to 50% (v/v) on carboxylesterase activity of selected polyesterases. Reaction mixtures contained 1 µg of purified enzyme in 50 mM CHES buffer (pH 9.0) with 1 mM pNP-octanoate as substrate and were incubated for 20 min at 30 °C. Solvents (0% to 50% (v/v): DCM, dichloromethane; DMSO, dimethyl sulfoxide; HPF, 1,1,1,3,3,3-hexafluoro-2-propanol. All assays were carried out in triplicate. Note: assays of ABO_2249 vs. DMSO and ALC24_1328 vs. ethanol were conducted with enzyme samples derived from separate purification batches

In presence of HFP all enzymes retained less than 5% of their initial activity. Similar negative effect was observed for DCM (Fig. 7), with complete loss of the activity with more than 10% of DCM in reaction mixture. DMSO overall had a positive effect on the screened enzymes. ABO_1197, ABO_1251 and ABO_2249 showed 10–20% increase in the activity in presence of up to 20% DMSO, for APA_5 DMSO dramatically increased the activity, adding up to 50% of solvent in reaction mixture. Ethanol had differential effects on enzyme activity. Activities of ABO_1197 and ABO_1251 decreased even at 5% (v/v) ethanol in the reaction mixture, whereas activities of ABO_2244, APA_5, and ALC24_1328 increased. For APA_5, the presence of 20% (v/v) ethanol resulted in an 84% increase in activity.

ABO_2249, APA_5, and ALC24_1328 showed relative tolerance to the polar solvents of low polarity, increasing sensitivity with the increase of solvents’ polar index (DMSO < ethanol < HFP). Fifty percent of DMSO retained a positive effect on these enzymes, though for ethanol, less than 1% of activity was retained at 50% reaction mixture solution.

### Combined effects of solvents and temperature on the enzymatic polyester depolymerisation

**Fig. 8:**
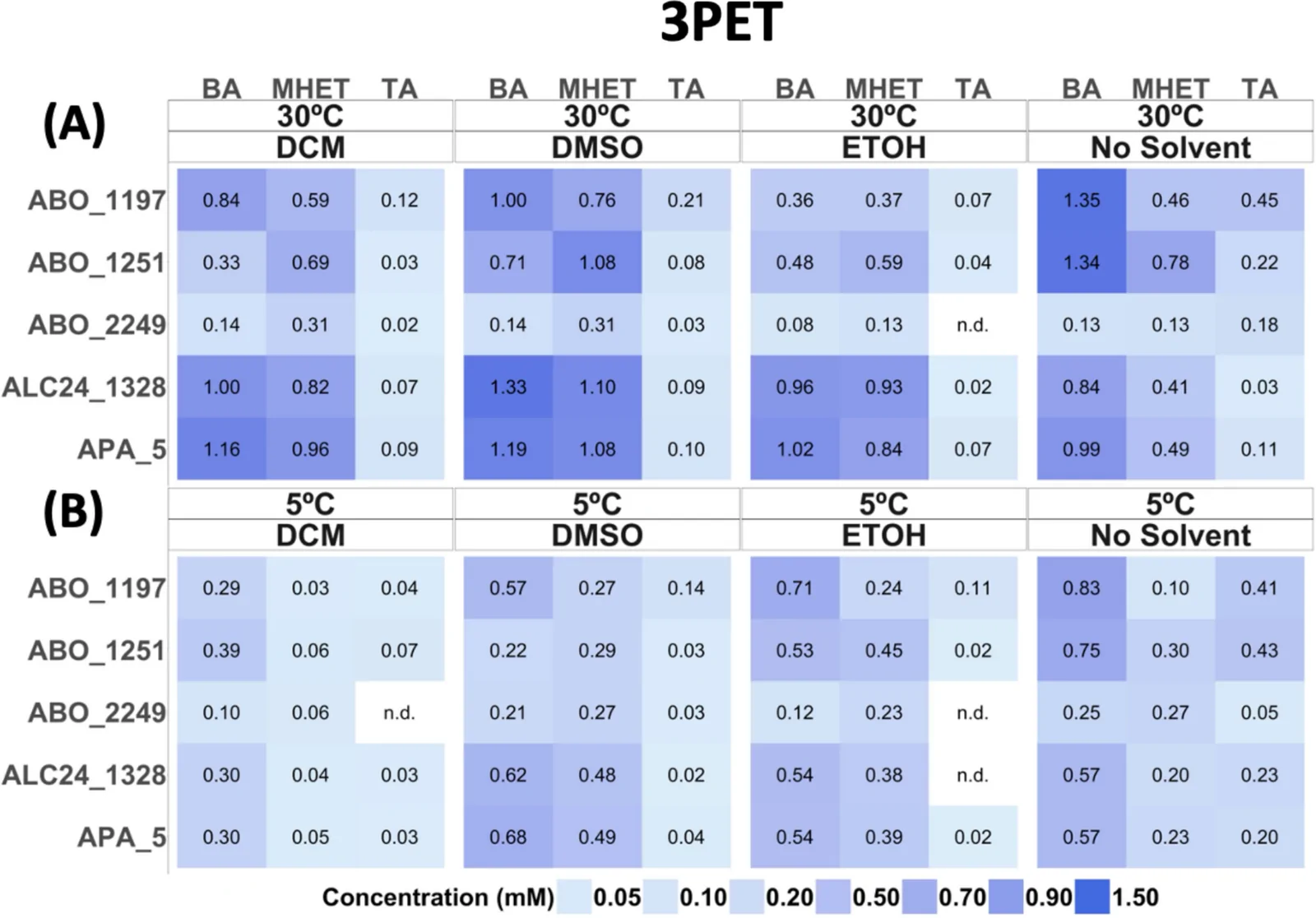
Effects of solvents and incubation temperature on 3PET degradation: HPLC analysis of reaction products. Reaction mixtures contained 50 µg of purified enzyme in 50 mM CHES buffer (pH 9.0) with the indicated solvents and were incubated overnight at 30 °C (**A**) and 5 °C (**B**). Reaction products (BA, MHET, and TA) were analysed by HPLC as described in the ‘Materials and methods’ section. ‘n.d.’, not detectable (below the detection limit of 0.01 mM). All assays were carried out in triplicate

In this study, selected enzyme-maintained activity at low temperatures 5–40 °C (Fig. 5B). ABO_2249 and APA_5 exhibited the highest cold adaptation, with activities maintained at 70–85% at 5 °C, compared to those at 30 °C. We tested our enzymes’ activity with model substrate *p*NP-hexanoate, in the presence of DMSO and ethanol and observed an improvement in the activity of up to 25 to 100% with the presence of 10–20% (v/v) for APA_5 and ALC24_1328. DCM and HFP showed a stronger inhibiting effect on target enzymes’ activity (Fig. 7). Based on these results, we selected 20% ethanol and DMSO to increase enzyme activity and 5% for DCM aiming to decrease the crystallinity of the substrate in the reaction mixture with actual polymeric substrates: 3PET, PCL14, and PDLLA. The incubations took place at 5 °C and 30 °C to make later comparisons. The reaction mixtures were filtered (10-kDa spin filters) and analysed by HPLC, the concentration of end products (mM) was used as a standard to define the depolymerisation efficiency (Fig. 8). Incubation of APA_5 and ALC24_1328 with 3PET at 5 ºC in the presence of DMSO increased the production of BA and MHET. For the rest of the enzymes (ABO_1197, ABO_1251, and ABO_2249), any solvent addition at 5 °C resulted in a decrease in depolymerisation efficiency.

**Fig. 9:**
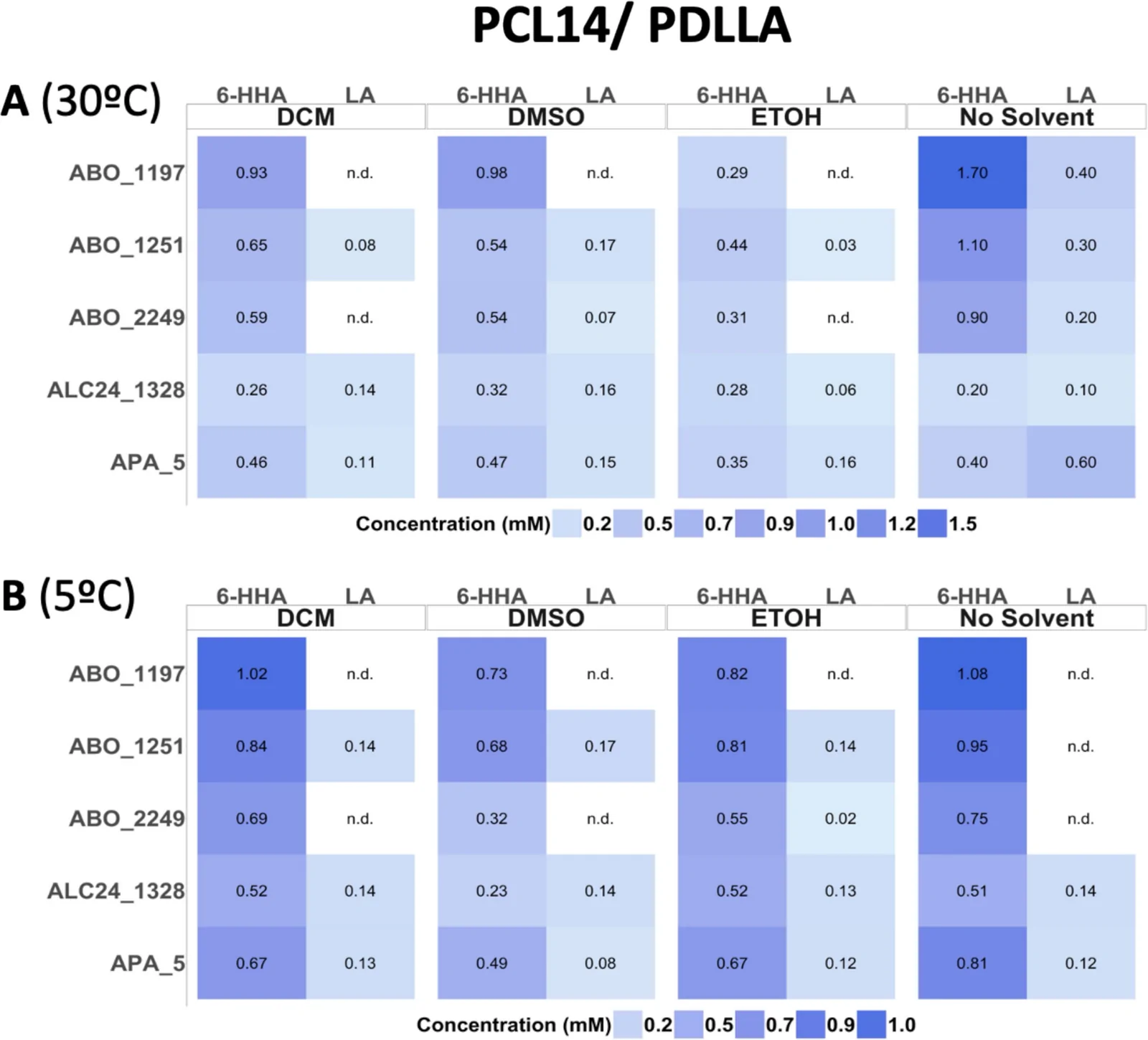
Effects of solvents and incubation temperature on the degradation of PCL14 and PDLLA: HPLC analysis of hydrolysis products. PCL14 and PDLLA were incubated with 50 µg of enzyme per reaction in three different organic solvents (DCM, DMSO, and ethanol (EtOH)) overnight at 30 °C (**A**) and 5 °C (**B**), and the formation of reaction products (6-HHA and LA) was analysed using HPLC as described in the ‘Materials and methods’ section. ‘n.d.’, not detectable (below the detection limit of 0.01 mM). All assays were carried out in triplicate

Addition of organic solvents DMSO, ethyl alcohol, and DCM at 30 °C slightly improved the depolymerisation efficiency for reactions with APA_5 and ALC24_1328, with both 3PET and PDLLA (Figs. 8, 9) when compared with no solvent conditions. Interestingly, even accounting for the protein activity inhibition at 5% DCM (Fig. 8), the increase in product concentration for 3PET depolymerisation for APA_5 and ALC24_1328 was observed at 30 °C; however, at 5 °C, the DCM addition decreased the yield of monomeric products threefold, as compared with 30 °C. At the same time, if accounting only for the temperature effect on the enzyme activity, more than twofold reduction in reaction products accumulation was observed in samples without solvent. The DCM has higher solubility in water at 5 vs. 30 °C (U.S. National Institute of Standards and Technology (NIST), Solubility Database, SRD 106, https://srdata.nist.gov/solubility/); therefore, at lower temperatures, the non-polar interaction with protein structures must increase, potentially causing negative structural changes in proteins. DCM showed improved results for 3PET depolymerisation for APA_5 and ALC24_1328 only at 30 °C.

### Structural analysis of five Alcanivoracaceae α/β-hydrolases with polyesterase activity

**Fig. 10:**
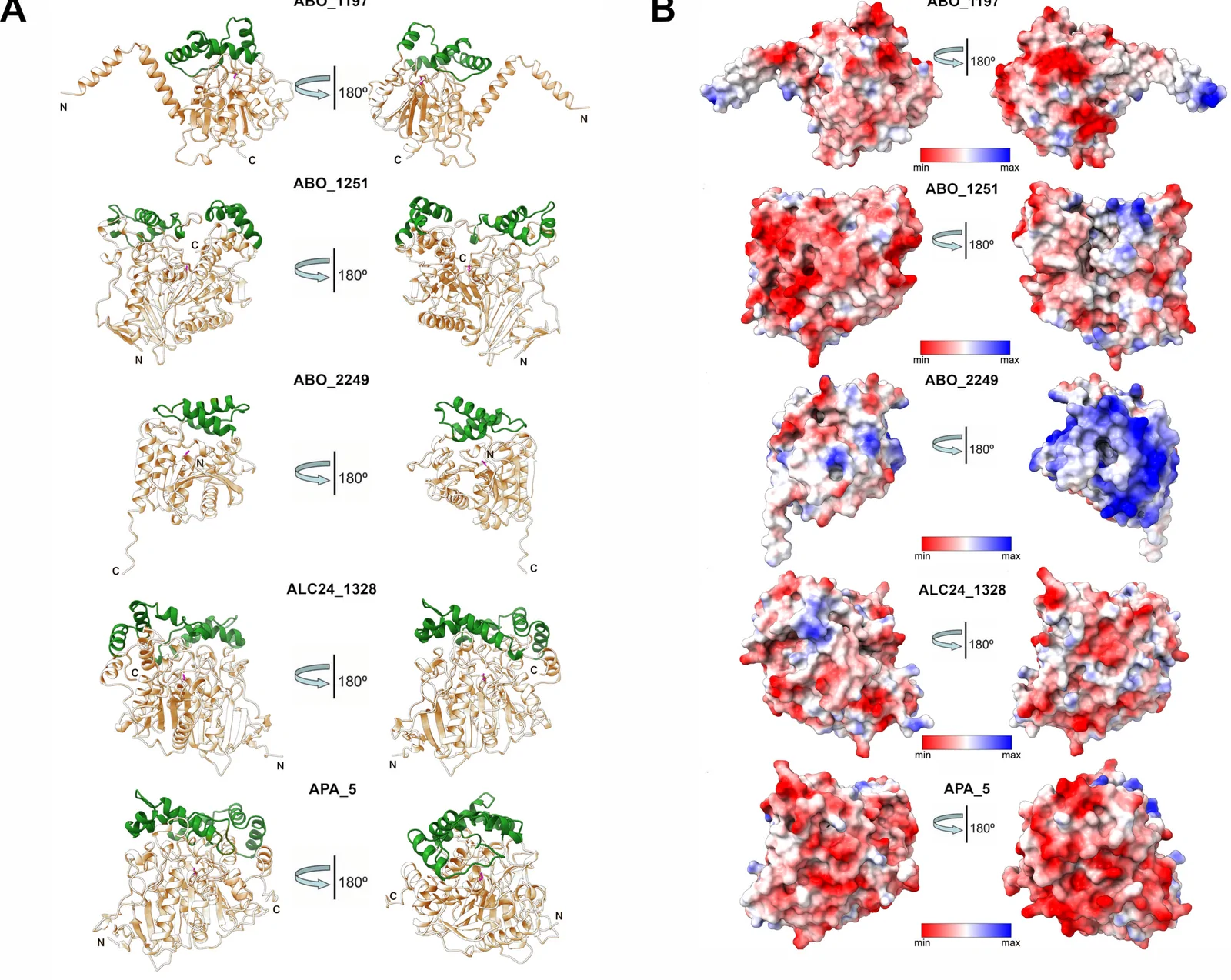
Structural models of polyesterases derived from *Alcanivoracaceae* genomes, ABO_1197, ABO_1251, ABO_2249, ALC24_1328, APA_5. **A** Overall folds and domain organisation, core domains coloured wheat and the lid domains coloured light green. Active site catalytic serine is coloured magenta. **B** Surface potential (Coulombic charge) distribution showing the electrostatic potential of solvent-accessible residues. The distribution is shown as a gradient from blue (positively charged) to red (negatively charged)

Structural models of esterases were generated using AlphaFold2 and revealed the presence of two domains: a core domain with a classical α/β hydrolase fold comprising 5–6 α-helices and a small, mostly helical lid domain (Fig. 10A). While the core domains of ABO_1197 and ABO_2249 contain N-terminal and C-terminal extensions, respectively, no extensions were observed in the core domains of other *Alcanivoracaceae* esterases. Early structural studies of PET-degrading hydrolases, including IsPETase and LCC, demonstrated that these enzymes lack lid domains, suggesting that the absence of a lid may be a prerequisite for efficient PET hydrolysis (Austin et al. 2018; Sulaiman et al. 2014). However, subsequent studies revealed the presence of a lid domain in several metagenomic and microbial PETases, notably in the archaeal PET46, which exhibits comparable activity on semi-crystalline PET powder (Erickson et al. 2022; Perez-Garcia et al. 2023). The core domains of *Alcanivoracaceae* enzymes also accommodate the active site with the catalytic Ser-His-Asp triad located below the lid domain (Fig. 10A, SI Fig. 1). Surface analysis of the electrostatic charge distribution of *Alcanivoracaceae* esterases revealed a predominance of negative charge in all proteins, except for ABO_2249, whose surface was mostly positively charged (Fig. 10B). This aligns with previous research suggesting that acidic (negatively charged) protein surfaces may help maintain hydration shells and the solubility of cold-active esterases at low temperatures (Noby et al. 2021). However, the distribution of surface hydrophobicity was similar among the five analysed *Alcanivoracaceae* enzymes, which were mostly hydrophilic, with small hydrophobic patches located near the active sites (SI Fig. 6). This is consistent with previous studies showing that a high abundance of hydrophilic residues on protein surfaces may be beneficial for enzyme activity at low temperatures (Nowak and Otzen 2024).

## Discussion

In this study, eleven α/β-hydrolases from *Alcanivoracaceae* were purified and biochemically characterised for their carboxylesterase and polyesterase activities, including six previously reported enzymes (Hajighasemi et al. 2016, 2018; Tchigvintsev et al. 2015), which were included as reference proteins (ABO_0116, ABO_1197, ABO_1251, ABO_1483, ABO_1895, and ABO_2249). All purified proteins exhibited carboxylesterase activity toward chromogenic *p*NP-esters with a broad substrate range and a preference for short-chain acyl esters, which is typical for carboxylesterases (Martínez-Martínez et al. 2018) (Hajighasemi et al. 2016, 2018; Tchigvintsev et al. 2015).

HPLC-based assays with emulsified polyesters revealed detectable activity in all enzymes against PCL2 and the model PET-like substrate 3PET (Fig. 3). Six enzymes also hydrolysed PBA, five proteins degraded PCL14 and PDLLA, while no activity was observed with other tested polyesters aPET, PLLA, PDLA, PBS, PC, and PBT. These results confirm and extend our recent observations that polyesterase activity is widespread in carboxylesterases from families IV and V (Ma et al. 2025). Moreover, our findings reinforce that HPLC-based assays provide greater sensitivity than agarose clearing methods for detecting polyesterase activity in purified proteins (Ma et al. 2025). The *Alcanivoracaceae* enzymes preferentially degraded aliphatic polyesters with shorter chain lengths and lower molecular weights (e.g. PCL2, PCL14, PDLLA, and PBA), while aromatic and more crystalline polyesters such as PC, PBT, and aPET were largely recalcitrant. This trend is consistent with the known influence of polymer chain length and crystallinity on enzymatic susceptibility, as longer and more crystalline polymers exhibit stronger intermolecular interactions and reduced chain mobility, requiring higher activation energy for hydrolysis. From an industrial perspective, cold-adapted enzymes offer distinct advantages, including reduced energy costs and protection of thermolabile products and intermediates (Santiago et al. 2016). However, because polyester crystallinity remains high at low temperatures, appropriate pretreatments to reduce crystallinities will likely be required to enable complete depolymerization with cold-adapted enzymes).

Marine microorganisms represent an important reservoir of cold-active and cold-tolerant enzymes due to the predominantly low temperatures of seawater habitats (Médigue et al. 2005; Kube et al. 2013; Santiago et al. 2016). For instance, EstB from *Alloalcanivorax dieselolei* displayed optimal activity around 20 °C and retained over 95% of activity between 0 and 10 °C (Degli-Innocenti et al. 2023; Zhang et al. 2014). Similarly, RhLip from *Rhodococcus* sp. AW25M09 retained 50% activity at 10 °C (Hjerde et al. 2013), and a carboxylesterase from *Psychrobacter cryohalolentis* maintained over 90% of its maximal activity at 0–5 °C (Novototskaya-Vlasova et al. 2012). The carboxylesterase OLEI01171 from *Oleispira* *antarctica* RB-8, another marine hydrocarbon degrader*,* also exhibits high activity between 5 and 30 °C (Lemak et al. 2012). Structural analyses of enzymes from *O. antarctica* RB-8 and their mesophilic homologues suggested that increased active site flexibility contributes to the catalytic efficiency of cold-active enzymes, compensating for the reduced thermal energy in cold environments (Kube et al. 2013). Consistent with this, all enzymes characterised in this study were active and stable at low temperatures (5–20 °C), with melting temperatures (*T*_m_) ranging from 30 to 48 °C. Lower *T*_m_ values were generally associated with enhanced cold tolerance, whereas higher *T*_m_ values correlated with increased thermostability. The enhanced cold tolerance observed in ABO_1197 and ABO_1251 may result from increased conformational flexibility conferred by residues such as Gly, Ser, and Met, and a lower abundance of Pro and Arg residues, which typically constrain structural mobility (Parvizpour et al. 2021). However, comparative sequence analysis of cold-active and thermostable esterases did not reveal a consistent correlation between amino acid composition and cold tolerance.

Except for APA_5, the *Alcanivoracaceae* polyesterases (ABO_1197, ABO_1251, ABO_2249, and ALC24_1328) exhibited high tolerance to, or partial activation in the presence of Tween 20 (Fig. 5A), a feature frequently observed among cold-adapted α/β-hydrolases. For instance, the three thermophilic metagenomic esterases showed no activation and low tolerance to detergents (Distaso et al. 2023), whereas several cold-adapted carboxylesterases from marine bacteria and a cold-active protease from *Psychrobacter* sp. 94-6 PB were stimulated by the addition of detergents, including Tween 20 (Novototskaya-Vlasova et al. 2012; Wu et al. 2013; Perfumo et al. 2020). Based on the structural analysis of the *Alcanivoracaceae* polyesterases (Fig. 10B, SI Fig. 6), the detergent tolerance of these enzymes appears to correlate with the abundance of hydrophilic and negatively charged residues on their surfaces.

Organic solvents can enhance enzymatic transformations by increasing substrate solubility and modulating enzyme flexibility via alterations in hydrogen bonding (Osbon and Kumar, 2019; Sorgenfrei et al. 2024). However, this simultaneously requires a certain level of enzyme tolerance to organic solvents. Given that *Alcanivoracaceae* species thrive in oil- and hydrocarbon-rich environments (Sabirova et al. 2006; Zhang et al. 2014), their enzymes are expected to exhibit tolerance to both low temperatures and organic solvents. Nevertheless, the inherent structural flexibility of cold-adapted enzymes may render them more susceptible to the destabilising effects of elevated temperatures and organic solvents (Sellek and Chaudhuri 1999). To date, only a limited number of cold-adapted esterases and lipases with solvent tolerance have been described (Lee et al. 2017). For instance, high solvent resistance was reported for the EstB esterase from *Alloalcanivorax dieselolei* B-5^T^ with the retention of 80% activity in the presence of isopropanol (70%) or acetone (up to 70%) (Zhang et al. 2014). In the presence of 50% dimethyl sulfoxide (DMSO) or ethanol, EstB retained 83% and 87% of activity, respectively, but it was strongly inhibited by acetonitrile and methanol (Zhang et al. 2014)). In the present study, the addition of DMSO up to 20% increased the carboxylesterase activity of all tested enzymes from *Alcanivoracaceae* (Fig. 7). Ethanol at 10–20% stimulated the activity of three enzymes but was inhibitory at higher concentrations, while DCM (dichloromethane) and HFP (hexafluoroisopropanol) caused strong inhibition. The addition of DMSO or ethanol enhanced 3PET depolymerisation by APA_5 and ALC24_1328 at 30 °C, whereas no stimulation was observed at 5 °C (Fig. 8). These findings suggest that, within the optimal range, higher reaction temperatures exert a more pronounced positive effect on enzymatic polyester depolymerisation than the presence of organic solvents.

## Conclusion

This study expands the current understanding of *Alcanivoracaceae* α/β-hydrolases by demonstrating that polyesterase activity is a common feature among their carboxylesterases, particularly within families IV and V. Eleven enzymes tested exhibited carboxylesterase activity with a preference for short-chain monoesters, as well as polyesterase activity toward aliphatic polyesters such as PCL and PBA, while showing limited activity toward aromatic polyesters. Their notable activity and stability at low temperatures, together with tolerance to detergents and certain organic solvents, reflect their adaptation to hydrocarbon-rich marine environments. These findings provide new insights into the biochemical diversity and ecological roles of *Alcanivoracaceae* enzymes and highlight their potential for environmentally friendly biotransformations, particularly in low-temperature, energy-efficient plastic biodegradation and recycling.

## Supplementary Information

Below is the link to the electronic supplementary material.

ESM 1 (DOCX 14.8 MB)

## Data Availability

No datasets were generated or analysed during the current study.

## Abbreviations

3PET: Bis(benzoyloxyethyl) terephthalate
PET: Poly(ethylene terephthalate)
aPET: Amorphous poly(ethylene terephthalate)
PU: Polyurethane
PE: Polyethylene
PS: Polystyrene
PC: Polycarbonate
PCL14: Polycaprolactone (*M*_n_ 14,000)
PCL2: Polycaprolactone (*M*_n_ 2000)
PBS: Polybutylene succinate
PBA: Polybutylene adipate
PBT: Polybutylene terephthalate
PLLA: Poly-L-lactide
PDLA: Poly-D-lactide
PDLLA: Poly-D,L-lactide
MHET: Mono(2-hydroxyethyl) terephthalate
BHET: Bis(2-hydroxyethyl) terephthalate
TA: Terephthalic acid
BPA: Bisphenol-A
DMSO: Dimethyl sulfoxide
DCM: Dichloromethane
HFP: 1,1,1,3,3,3-Hexafluoro-2-propanol
