# Supplementary material for: Cold-adapted carboxylesterases from Alcanivoracaceae active with a wide range of synthetic polyesters
Source: Appl Microbiol Biotechnol. 2026 Feb 7;110(1):59. doi: 10.1007/s00253-026-13726-z (PMC12886402; doi:10.1007/s00253-026-13726-z)
Supplement: Supplementary file 1 — (DOCX 14.8 MB) [file 253_2026_13726_MOESM1_ESM.docx]

**Supplementary Information**

For the manuscript: **Cold-adapted carboxylesterases from *Alcanivoracaceae* active with a wide range of synthetic polyesters**, co-authored by Hairong Ma et al.

**Content**

**Supplementary tables** Page

**SI Table 1. α/ β-fold hydrolases from Alcanivoracaceae characterised in this study.** 2

**SI Table 2. HPLC product analysis and plate assay results.** 3

**SI Table 3. Amino acid content in enzymes from this study and previously characterised (reference) enzymes** 4

**SI Table 4. Assays of α/β-hydrolases for carboxylesterase activity using chromogenic *p*NP-esters with varying acyl chain lengths (C2-C16)** 5

**Supplementary figures** 7

**SI Figure 1. Multiple sequence alignments of polyesterases** 7

**SI Figure 2. Neighbor-Joining phylogenetic tree of enzymes selected for this study** 10

**SI Figure 3. SDS-PAGE analysis of purified enzymes** 11

**SI Figure 4. Agarose-based screening of purified proteins for hydrolytic activity against various polyesters** 12

**SI Figure 5. DSF analysis** 13

**SI Figure 6. Structural analysis of surface hydrophobicity of the *Alcanivoracaceae*-derived polyesterases** 14

**SI Table 1. α/ β-fold hydrolases from Alcanivoracaceae characterised in this study.**

| **Protein name**  (this work) | **Accession** | **Organism/species** | **Signal peptide** | **Mr (kDa)** | **Clade location** |
| --- | --- | --- | --- | --- | --- |
| 1. AGE_3 | WP_138772051 | *Alloalcanivorax (Alcanivorax) gelatiniphagus* **MEBiC08158T** | - | 57.7 | IV |
| 2. AGE_8 | WP_138772014 | *Alloalcanivorax (Alcanivorax) gelatiniphagus* **MEBiC08158 T** | - | 32.8 | I |
| 3. AGE_12 | WP_138772717 | *Alloalcanivorax (Alcanivorax) gelatiniphagus* **MEBiC08158 T** | - | 32.9 | III |
| 4. APA_3 | WP_035204471 | *Isoalcanivorax (Alcanivorax) pacificus* W11-5 **T** | - | 64.0 | IV |
| 5. APA_4 | WP_008736028 | *Isoalcanivorax (Alcanivorax) pacificus* W11-5 **T** | - | 57.3 | IV |
| 6. APA_5 | WP_008737557 | *Isoalcanivorax (Alcanivorax) pacificus* W11-5 **T** | + | 55.0 | IV |
| 7. ALC24_1162 | WP_133490829 | *Alloalcanivorax (Alcanivorax)* sp. 24 | - | 35.2 | III |
| 8. AGE_2 | WP_138771864 | *Alloalcanivorax (Alcanivorax) gelatiniphagus* **MEBiC08158 T** | - | 21.8 | I |
| 9. APA_2 | WP_008737455 | *Isoalcanivorax (Alcanivorax) pacificus* W11-5 **T** | - | 33.2 | I |
| 10. APA_8 | WP_008737253 | *Isoalcanivorax (Alcanivorax) pacificus* W11-5 **T** | - | 33.5 | I |
| 11. ALC24_3989 | WP_133493397 | *Alloalcanivorax (Alcanivorax)* sp. 24 | - | 64.2 | IV |
| 12. ALC24_1328 | WP_133490973 | *Alloalcanivorax (Alcanivorax)* sp. 24 | - | 56.0 | IV |
| 13. ALC24_2069 | WP_133491672 | *Alloalcanivorax (Alcanivorax)* sp. 24 | - | 34.2 | III |
| 14. ALC24_4107 | WP_133493510 | *Alloalcanivorax (Alcanivorax)* sp. 24 | - | 65.2 | I |
| 15. APA_6 | WP_008739160 | *Isoalcanivorax (Alcanivorax) pacificus* W11-5 | + | 28.4 | IV |
| 16. ABO_0116 | Q0VTH9 | *Alcanivorax borkumensis* SK2 **T** | - | 31.2 | I |
| 17. ABO_1197 | Q0VQA3 | *Alcanivorax borkumensis* SK2 **T** | + | 35.1 | III |
| 18. ABO_1251 | Q0VQ49 | *Alcanivorax borkumensis* SK2 **T** | - | 54.6 | IV |
| 19. ABO_1483 | Q0VPG7 | *Alcanivorax borkumensis* SK2 **T** | - | 32.0 | I |
| 20. ABO_1895 | Q0VNA5 | *Alcanivorax borkumensis* SK2 **T** | + | 24.2 | I |
| 21. ABO_2249 | Q0VLQ1 | *Alcanivorax borkumensis* SK2 **T** | - | 32.6 | III |

**SI Table 2**. **HPLC product analysis and plate assay results.** “+/-“ indicates presence/absence of activity on agar plates with polymers’ suspensions. Numbers in brackets indicate average concentration (mM) of monomers quantified by HPLC after overnight incubation at 30 ºC in reaction mixtures of the total volume of 200 µl, 50 mM CHES pH 9.0, 50 µg enzyme/reaction, 50 µl of suspended polyester nanoparticles.

| **Enzyme name** | **Amino acids % in polypeptide** | | | | | **Reference** |
| --- | --- | --- | --- | --- | --- | --- |
|  | Gly | Ser | Met | Pro | Arg |  |
| HotPETase | 7.8 | 10.0 | 2.8 | 6.0 | 4.1 | Wang et al., 2024 |
| CBA10055 | 8.8 | 5.0 | 2.2 | 8.0 | 6.6 | Ma et al., 2025 |
| EstB (*A. dieselolei*) | 7.6 | 5.9 | 1.5 | 6.2 | 6.5 | Zhang et al., 2014 |
| Est97 | 13.1 | 5.8 | 3.9 | 4.6 | 5.8 | Fu et al., 2013 |
| ABO_1197 | 6.9 | 5.7 | 3.1 | 5.0 | 4.1 | Tchigvintsev et al., 2015 |
| ABO_1251 | 9.3 | 5.1 | 2.4 | 5.9 | 5.7 | Tchigvintsev et al., 2015 |
| ABO_2249 | 9.8 | 7.2 | 4.3 | 7.5 | 6.6 | Martínez-Martínez et al., 2018 |
| APA_5 | 11.8 | 4.1 | 1.9 | 6.8 | 7.8 | this study |
| ALC24_1328 | 10.6 | 4.9 | 2.8 | 7.5 | 8.7 | this study |

**SI Table 3. Amino acid content in enzymes from this study and previously characterised (reference) enzymes**

**SI Table 4.** **Assays of α/β-hydrolases for carboxylesterase activity using chromogenic pNP-esters with varying acyl chain lengths (C2-C16).**

| **Sample** | **Substrate** | **Activity (U/mg)** |
| --- | --- | --- |
| **ABO_0116** | *p*NP acetate (C2) | 4.30 ± 0.29 |
|  | *p*NP butyrate (C4) | 1.64 ± 0.17 |
|  | *p*NP octanoate (C8) | 0.67 ± 0.06 |
|  | *p*NP decanoate (C10) | 0.14 ± 0.02 |
|  | *p*NP dodecanoate (C12) | n.d. |
|  | *p*NP myristate (C14) | n.d. |
|  | *p*NP palmitate (C16) | n.d. |
|  | *p*NP acetate (C2) | 51.30 ± 4.70 |
| **ABO_1197** | *p*NP butyrate (C4) | 24.87 ± 4.60 |
|  | *p*NP octanoate (C8) | 1.82 ± 0.07 |
|  | *p*NP decanoate (C10) | 1.26 ± 0.01 |
|  | *p*NP dodecanoate (C12) | 1.03 ± 0.01 |
|  | *p*NP myristate (C14) | 0.163 ± 0.003 |
|  | *p*NP palmitate (C16) | 0.724 ± 0.002 |
|  | *p*NP acetate (C2) | 89.63 ± 5.41 |
|  | *p*NP butyrate (C4) | 103.29 ± 3.65 |
| **ABO_1251** | *p*NP octanoate (C8) | 1.77 ± 0.01 |
|  | *p*NP decanoate (C10) | 0.82 ± 0.03 |
|  | *p*NP dodecanoate (C12) | 0.71 ± 0.02 |
|  | *p*NP myristate (C14) | 0.14 ± 0.01 |
|  | *p*NP palmitate (C16) | 0.62 ± 0.02 |
| **ABO_1483** | *p*NP acetate (C2) | 0.80 ± 0.04 |
|  | *p*NP butyrate (C4) | 3.53 ± 0.19 |
|  | *p*NP octanoate (C8) | n.d. |
|  | *p*NP decanoate (C10) | 0.02 ± 0.01 |
|  | *p*NP dodecanoate (C12) | 0.04 ± 0.01 |
|  | *p*NP myristate (C14) | n.d. |
|  | *p*NP palmitate (C16) | 0.03 ± 0.02 |
| **ABO_1895** | *p*NP acetate (C2) | 1.44 ± 0.53 |
|  | *p*NP butyrate (C4) | 6.26 ± 1.72 |
|  | *p*NP octanoate (C8) | 1.21 ± 0.04 |
|  | *p*NP decanoate (C10) | 0.66 ± 0.03 |
|  | *p*NP dodecanoate (C12) | 0.14 ± 0.01 |
|  | *p*NP myristate (C14) | 0.022 ± 0.001 |
|  | *p*NP palmitate (C16) | 0.49 ± 0.05 |
| **ABO_2249** | *p*NP acetate (C2) | 22.11 ± 0.45 |
|  | *p*NP butyrate (C4) | 66.38 ± 1.35 |
|  | *p*NP octanoate (C8) | 2.35 ± 0.25 |
|  | *p*NP decanoate (C10) | 1.34 ± 0.05 |
|  | *p*NP dodecanoate (C12) | 1.05 ± 0.02 |
|  | *p*NP myristate (C14) | 0.232 ± 0.004 |
|  | *p*NP palmitate (C16) | 0.72 ± 0.04 |
| **ALC24_1328** | *p*NP acetate (C2) | 120.81 ± 4.70 |
|  | *p*NP butyrate (C4) | 80.72 ± 7.54 |
|  | *p*NP octanoate (C8) | 1.80 ± 0.08 |
|  | *p*NP decanoate (C10) | 1.23 ± 0.10 |
|  | *p*NP dodecanoate (C12) | 1.03 ± 0.13 |
|  | *p*NP myristate (C14) | 0.26 ± 0.06 |
|  | *p*NP palmitate (C16) | 0.67 ± 0.02 |
| **AGE_12** | *p*NP acetate (C2) | 2.84 ± 0.14 |
|  | *p*NP butyrate (C4) | 2.19 ± 0.23 |
|  | *p*NP octanoate (C8) | 3.32 ± 0.04 |
|  | *p*NP decanoate (C10) | 1.74 ± 0.15 |
|  | *p*NP dodecanoate (C12) | 1.30 ± 0.09 |
|  | *p*NP myristate (C14) | 0.93 ± 0.04 |
|  | *p*NP palmitate (C16) | 0.74 ± 0.03 |
| **APA_2** | *p*NP acetate (C2) | 2.07 ± 0.08 |
|  | *p*NP butyrate (C4) | 2.28 ± 0.31 |
|  | *p*NP octanoate (C8) | 0.99 ± 0.02 |
|  | *p*NP decanoate (C10) | 1.14 ± 0.09 |
|  | *p*NP dodecanoate (C12) | 3.03 ± 0.14 |
|  | *p*NP myristate (C14) | 0.64 ± 0.04 |
|  | *p*NP palmitate (C16) | 0.193 ± 0.003 |
| **APA_3** | *p*NP acetate (C2) | 0.88 ± 0.01 |
|  | *p*NP butyrate (C4) | 0.84 ± 0.19 |
|  | *p*NP octanoate (C8) | 0.43 ± 0.01 |
|  | *p*NP decanoate (C10) | 0.383 ± 0.004 |
|  | *p*NP dodecanoate (C12) | n.d. |
|  | *p*NP myristate (C14) | 0.580 ± 0.001 |
|  | *p*NP palmitate (C16) | 0.427 ± 0.002 |
| **APA_5** | *p*NP acetate (C2) | 144.64 ± 4.31 |
|  | *p*NP butyrate (C4) | 176.74 ± 2.61 |
|  | *p*NP octanoate (C8) | 1.97 ± 0.08 |
|  | *p*NP decanoate (C10) | 0.94 ± 0.22 |
|  | *p*NP dodecanoate (C12) | 0.88 ± 0.15 |
|  | *p*NP myristate (C14) | 0.22 ± 0.05 |
|  | *p*NP palmitate (C16) | 0.71 ± 0.06 |

* Values are expressed as mean ± SD (n=3).

A)A)


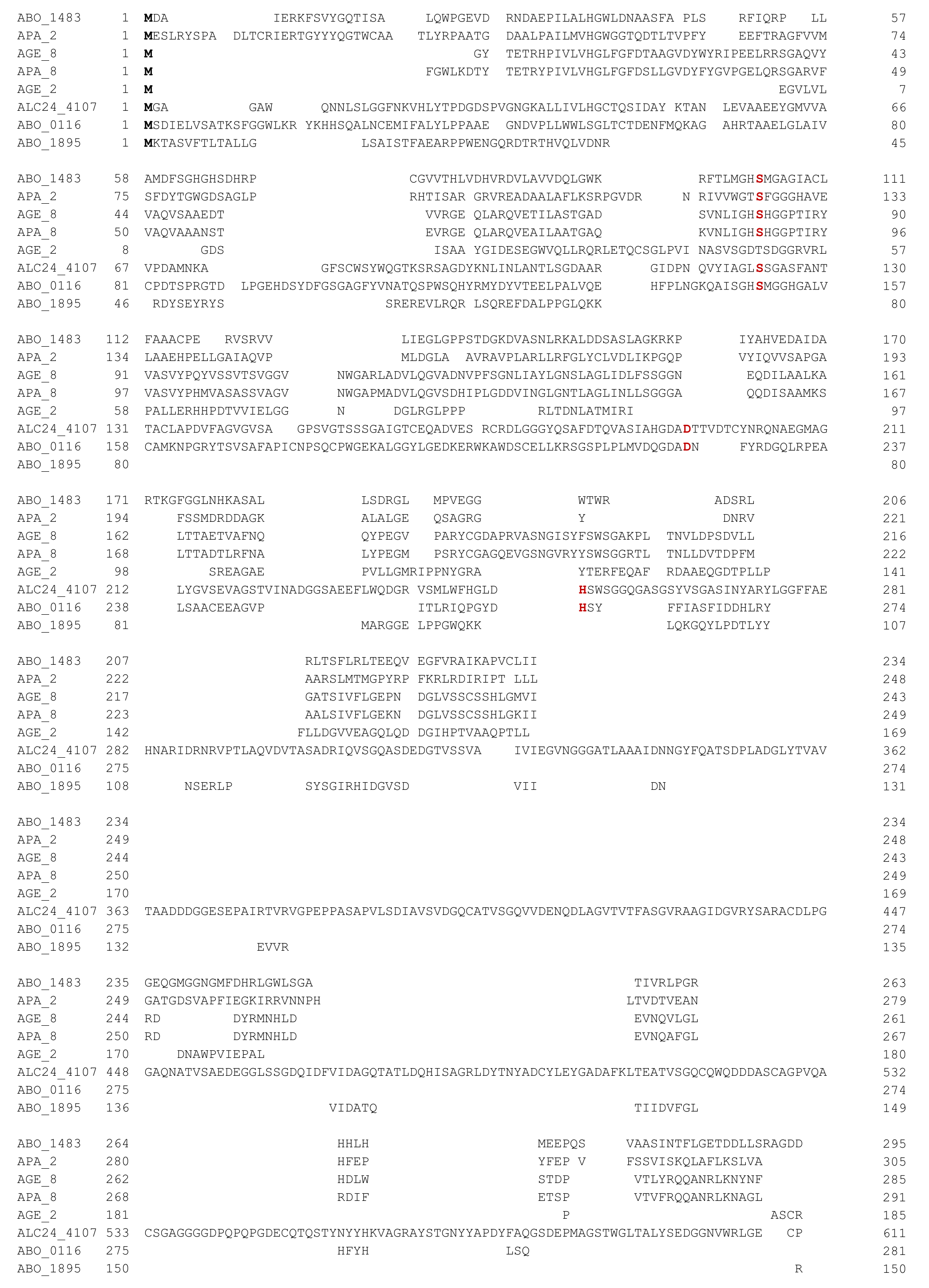


A)B)


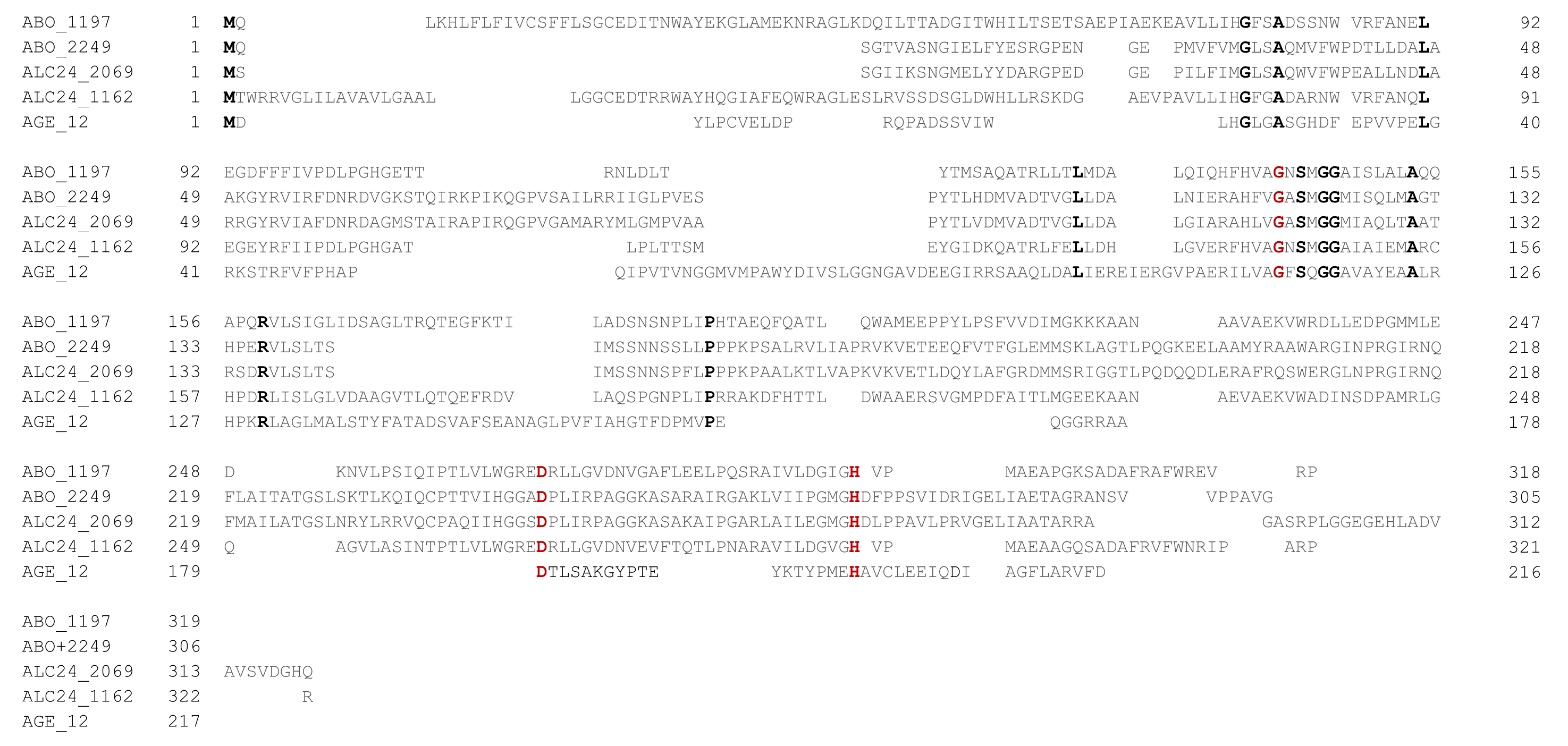


C)


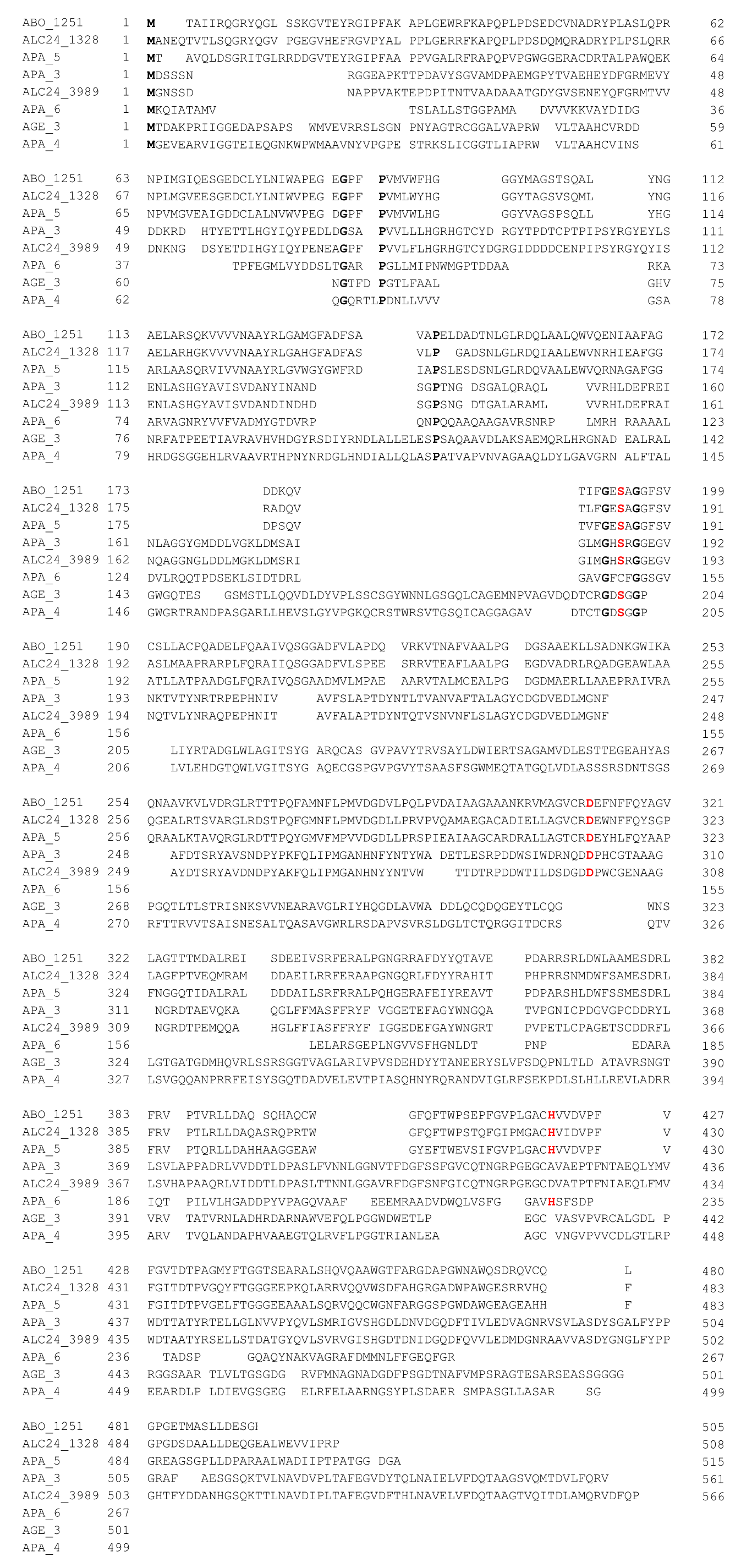


**SI Figure 1.** **Multiple sequence alignments of polyesterases**. Cluster I **(A),** Cluster III **(B),** and Cluster IV **(C)**. Catalytic triad residues (Ser, Asp, and His) are highlighted in red among the conserved positions (bold). See Fig. 1 for reference.


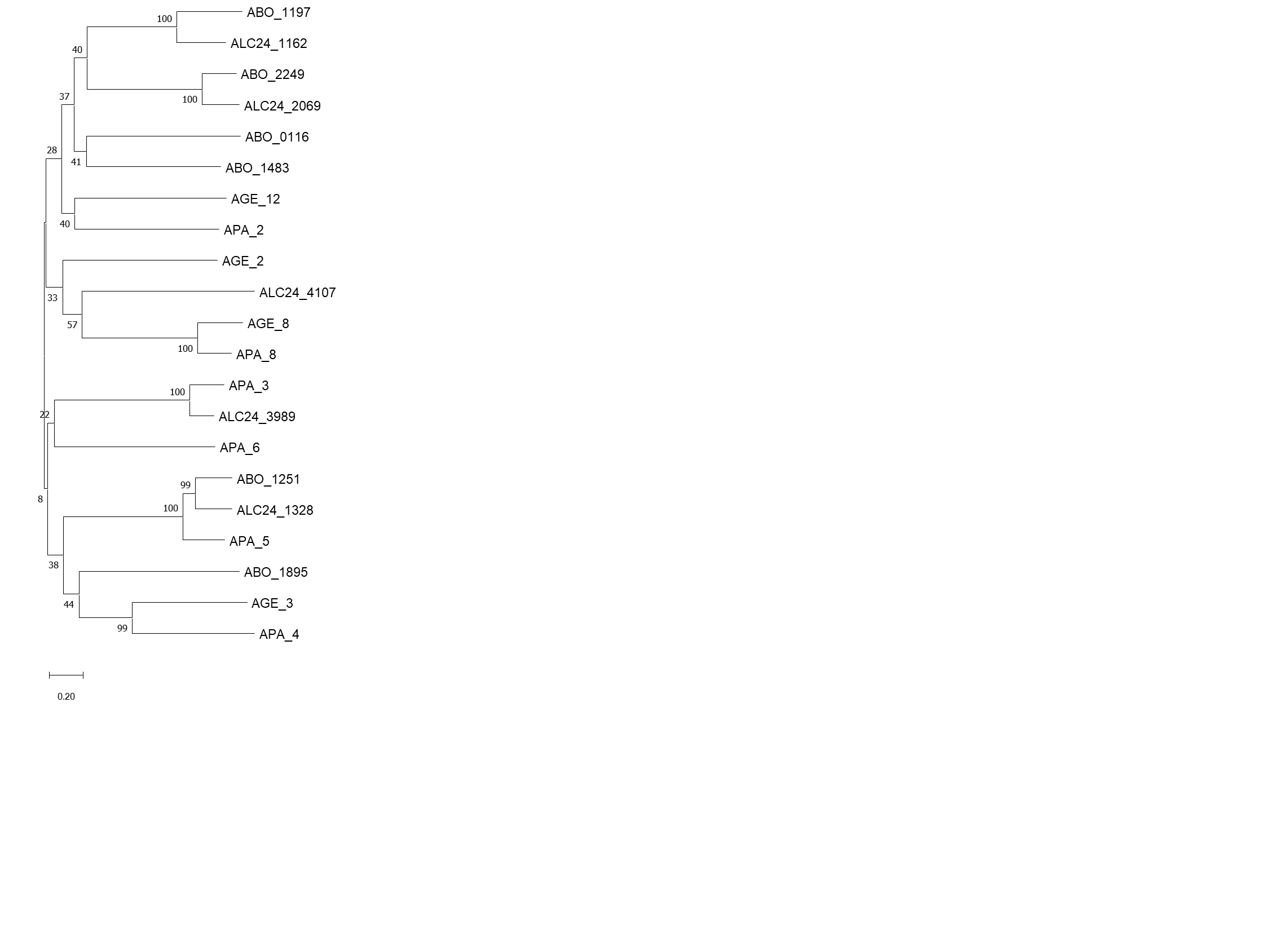


**SI Figure 2.** **Neighbor-Joining phylogenetic tree of enzymes selected for this study.** All ambiguous positions were removed for each sequence pair (pairwise deletion option). There were a total of 751 positions in the final dataset. Protein sequence alignment using Muscle alignment tool and Neighbor-Joining phylogeny test of 500 pseudoreplicates were conducted in MEGA11 (Kumar et al, 2021). Bootstrap values are shown at branching points. Scale bar, 0.2 substitutions per position.

**
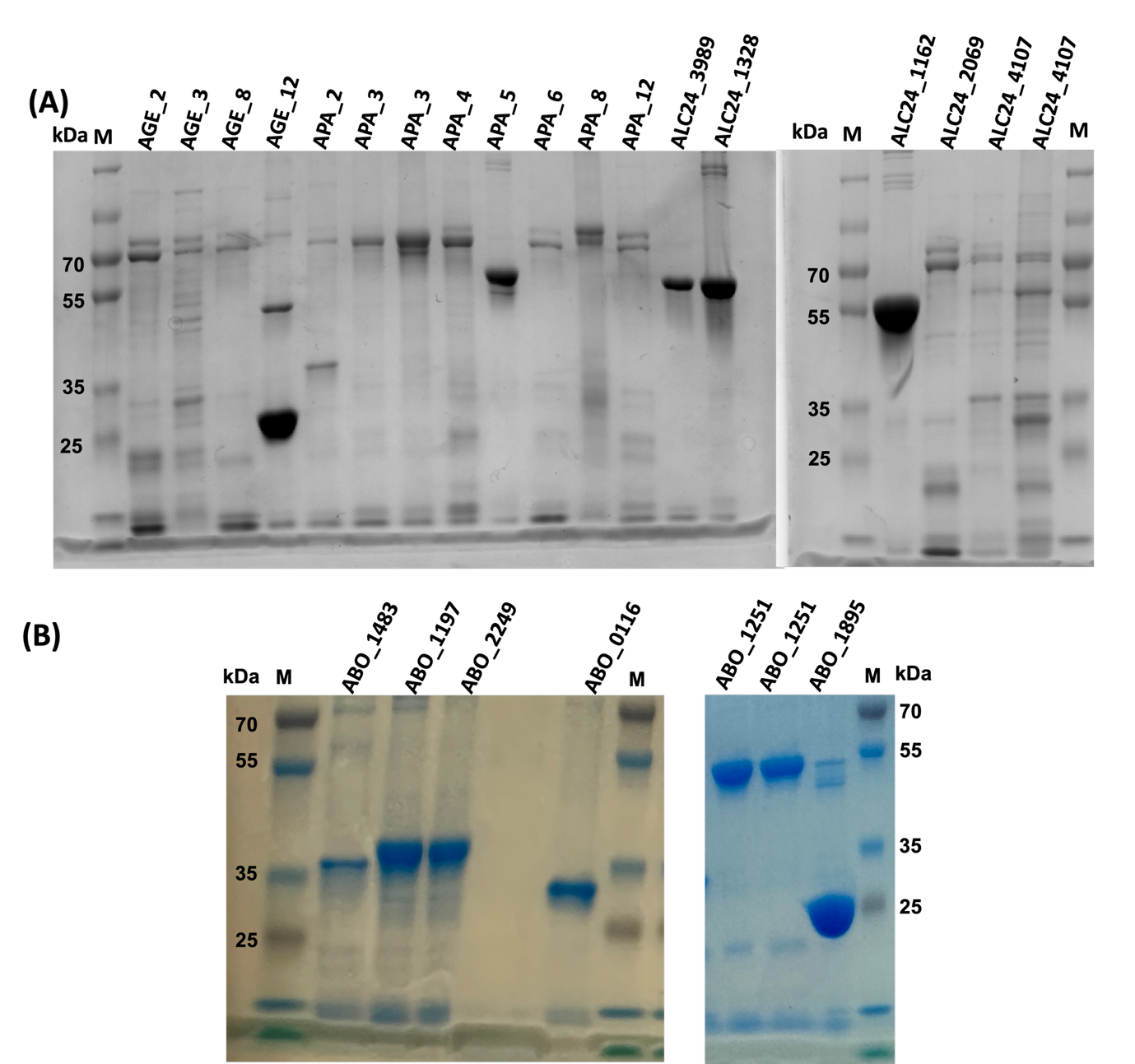
**

**SI Figure 3. SDS-PAGE analysis of purified enzymes.** 6His-tagged proteins were recombinantly expressed in E. coil LOBSTR cells and affinity-purified using nickel-chelate chromatography followed by SDS-PAGE analysis of purified protein (2 µg/well) and Coomassie staining. M, protein molecular weight maker (PageRulerTmPlus Prestained Protein Ladder, Themo Scientific). (A) 11 esterases from Isoalcanivorax pacificus (APA), Alloalcanivorax sp. 24 (ALC24), and Alloalcanivorax gelatiniphagus (AGE), (B) 6 esterases from A. borkumensis SK2 (ABO).

**SI Figure 4.** **Agarose-based screening of purified proteins for hydrolytic activity against various polyesters.** Plates/polyesters: (A), PDLLA; (B), IMPRANIL; (C), PCL14; (D), aPET; (E), 3PET; (F), PCL2; (G), PBA; (H), PC; (I), PLLA; (J), PDLA; (K), PBS. The presence of activity is indicated by the formation of a clear zone around the wells loaded with indicated proteins (50 µg/well, 72 h at 30 ºC). Proteins: (1), AGE_12; (2), APA_5; (3), APA_3; (4), APA_5; (5), ALC24_1328; (6), ABO_0116; (7), ABO_1197; (8), ABO_1483; (9), ABO_1251; (10), ABO_1895; (11), ABO_2249; (12), -VE. The wells labelled “-VE” represent negative controls (no protein).


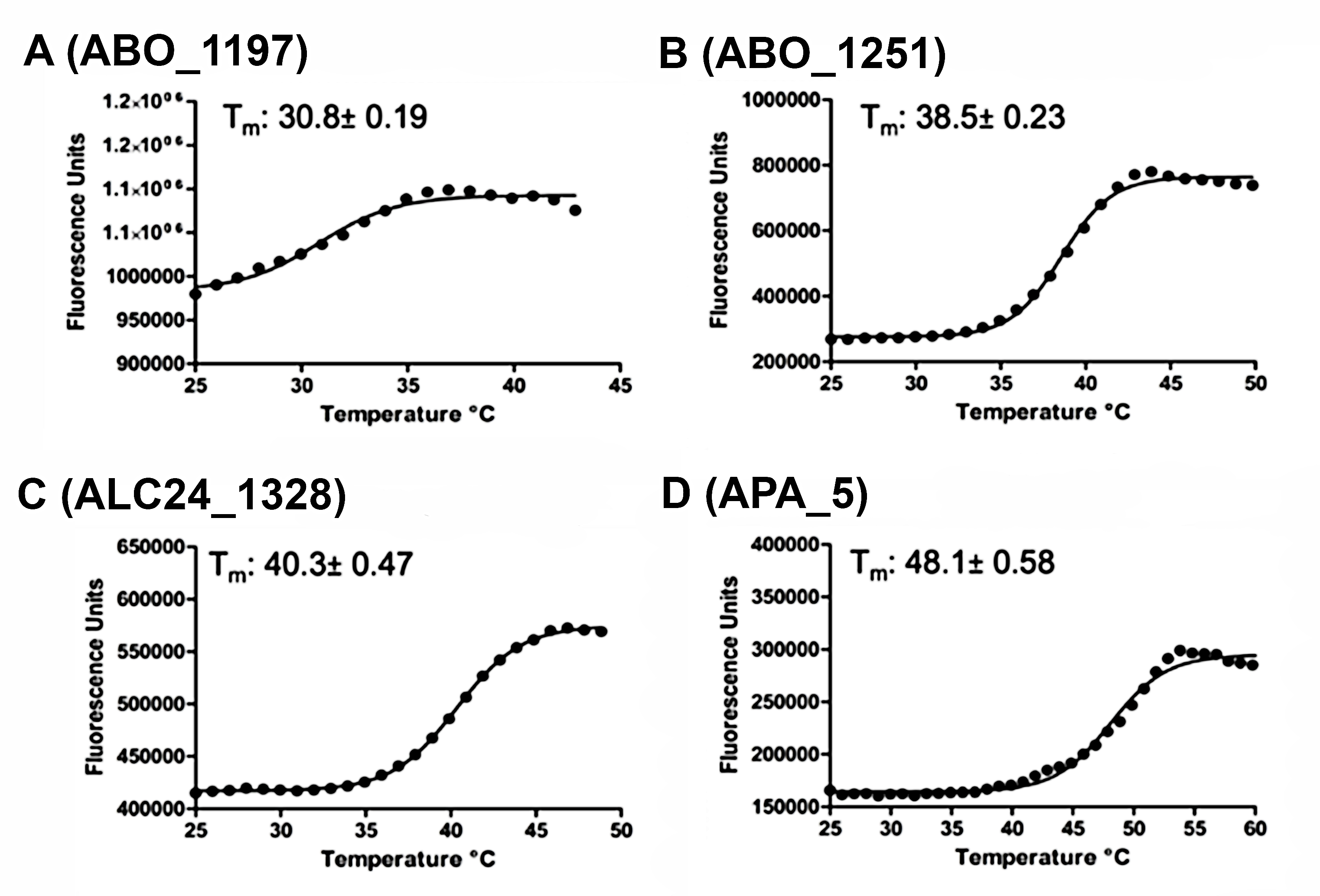


**SI Figure 5.** **DSF analysis.** Reaction conditions:10 µg of protein in 50 mM CHES buffer (pH 9.0 CHES), 25X SYPRO Orange, emission 300 nm, excitation 472 nm. (Low quality of the curve for ABO_2249 is not presented.


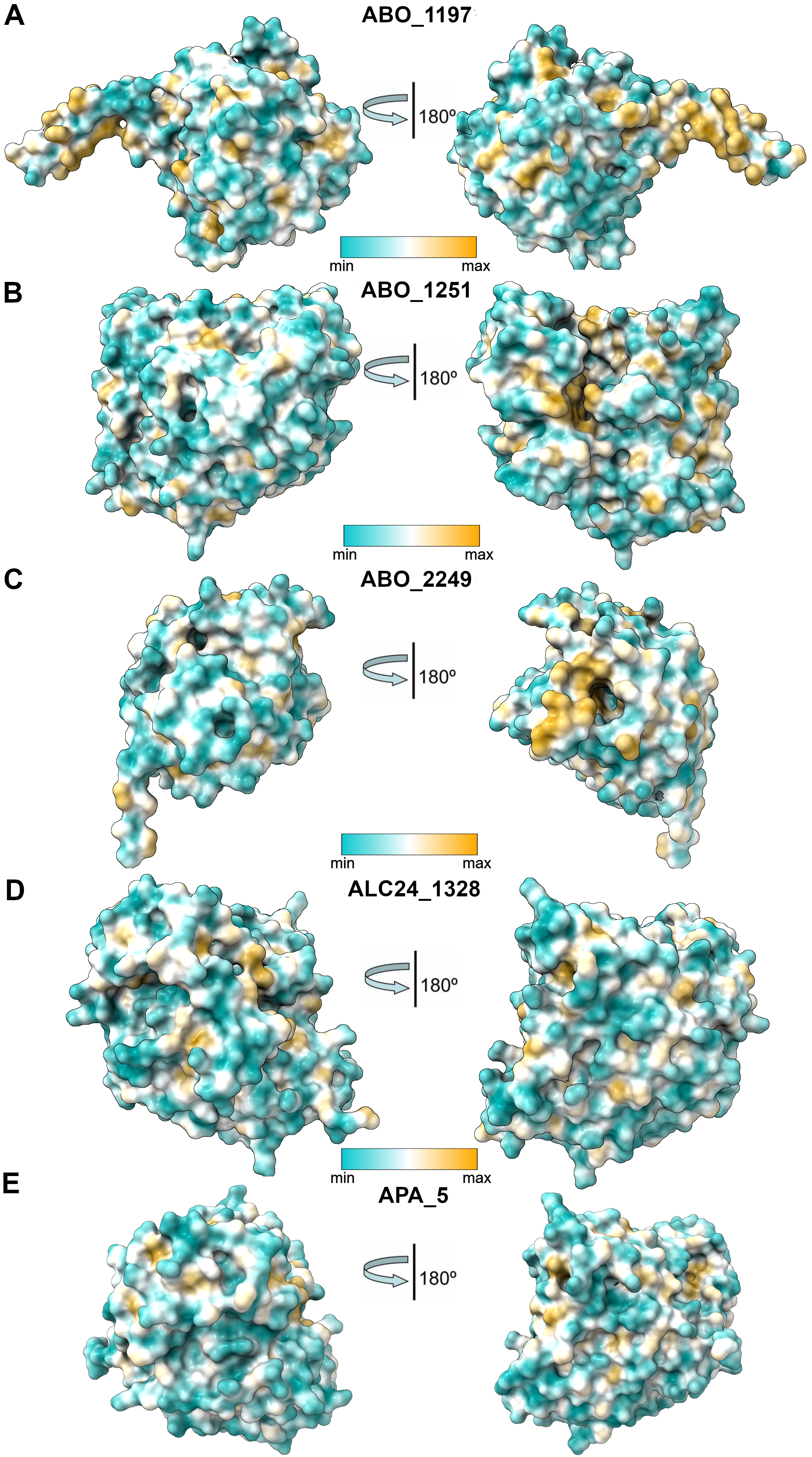


**SI Figure 6. Structural analysis of surface hydrophobicity of the *Alcanivoracaceae-*derived polyesterases.** Surface presentations of ABO_1197 (**A**), ABO_1251 (**B**), ABO_2249 (**C**), ALC24_1328 (D) and APA_5 (E) showing the hydrophobicity of solvent-accessible protein residues. The distribution of surface hydrophobicity is shown as a gradient from cyan (polar) to yellow (hydrophobic).
